# DUF4004 protein BacR (YhbD) of *Bacillus subtilis* is a regulator of bactofilin expression and interacts with bactofilins in a switch-like manner

**DOI:** 10.3389/fmicb.2026.1922879

**Published:** 2026-09-25

**Authors:** Kristin Velten, Patrik Brück, Shahla Alipour, Doreen Meier, Georg K. A. Hochberg, Paul Klemm, Marcus Lechner, Anke Becker, Peter L. Graumann

**Affiliations:** 1 SYNMIKRO, Center for Synthetic Microbiology, Philipps-University Marburg, Marburg, Germany; 2 Department of Chemistry, Philipps-University Marburg, Marburg, Germany; 3 Department of Biology, Philipps-University Marburg, Marburg, Germany

**Keywords:** *Bacillus subtilis*, bactofilins, cell wall stress, DUF4004, subcellular relocalization, transcriptional regulation

## Abstract

**Introduction:**

DUF4004-containing proteins are found in a variety of bacteria. The only characterized member was shown to act as transcriptional repressor of genes involved in metabolism in *Clostridium acetobutylicum*. We have characterized the *B. subtilis* member of this protein family, encoded by the *yhbD* gene. The absence of *yhbD* negatively affects the expression of the *yhbDEF* operon, and thereby of bactofilin genes, but does not show any other alteration of genome-wide transcript levels.

**Results:**

We propose to name the protein as “BacR” for bactofilin regulator. The putative transcription factor binds to DNA *in vitro*, and forms predominantly dimers. Interestingly, purified bactofilins BacE and BacF bind to BacR *in vitro*, and strongly reduce its DNA binding, and the depletion of bactofilins leads to reduced copy numbers of BacR. *In vivo*, BacR localizes in foci at the cell membrane.

**Discussion:**

Depletion of BacE and BacF leads to a loss of membrane localization of BacR and induces nucleoid association. These findings suggest that bactofilins can recruit BacR to the membrane. Treatment of cells with vancomycin also leads to a switch in BacR localization to the nucleoids, indicating that it may be involved in the response to cell wall stress. Our data suggest that BacR acts as a regulator of bactofilins, and fine-tunes bactofilin expression involving a switch-like mechanism with BacE and BacF.

## 1 Introduction

The regulation of transcription by transcription factors (TFs) has many facettes, ranging from long-known metabolite-induced changes converting active to inactive TFs (or vice versa), to phosphorylation-mediated activation/inactivation of DNA binding, or proteolytic activation (Browning et al., 2019). TF activity can also be linked to spatial cues, e.g., release from specific binding to a membrane transporter to be able to find binding sites near promoters on the nucleoids (Bohm and Boos, 2004). Thus, regulation of gene expression employs practically all kinds of biophysical aspects, from chemical modification to spatial sequestration by subcellular localization.

Especially for model bacteria, extensive studies of genome-wide transcriptional regulation are available, such that more and more, genetic network interactions become apparent, explaining many known pathways (Balleza et al., 2009). However, amongst genes of unknown function, many are predicted to be TFs, but their contribution to genetic networks in still unclear. This is true for the family of proteins containing domain of unknown function (DUF) 4004, which includes one member known to be a TF in *Clostridium acetobutylicum* called Cap0037. Cap0037 is a self-regulatory protein acting as a global TF, regulating the expression of 258 genes and thereby significantly affecting the metabolism of *C. acetobutylicum* under both acidogenic and alcohologenic fermentation conditions (Janssen et al., 2010; Nguyen et al., 2016). Affected genes included members of the Fur regulon involved in iron transport (*CA_C1029–CA_C1032*) and a flavodoxin-encoding gene. Cap0037 was shown to bind to its own promoter driving an operon (on the pSOL1 mega-plasmid) that includes *adhE2*, an aldehyde and alcohol dehydrogenase, but also to the *sol* operon and to *adc* promoter regions (Nguyen et al., 2016). It has been unclear if other members of the protein family also act as TFs, and likewise on a global genomic scale.

We set out to characterize the product of the *yhbD* gene in *Bacillus subtilis*, a close homolog of Cap0037, as second example from the DUF4004 protein family. The *cap0037* gene is situated upstream of a bactofilin-encoding gene, which is true for some other relatives (Supplementary Figure S1A). In *B. subtilis, yhbD* is upstream of two bactofilin genes, *yhbE* and *yhbF* (Figure 1), encoding for bactofilins BacE and BacF. Note that these names are also used for bacilysin synthesis and secretion proteins, wherefore we are maintaining the “*yhb*” gene annotation. This genetic context is conserved in several species (Supplementary Figure S1A), indicating a possible connection between DUF4004 proteins and bactofilins. The latter form filamentous structures in a variety of Gram positive and Gram negative bacteria, and perform a number of different cellular tasks (Liu et al., 2025). In *Caulobacter crescentus* and *Helicobacter pylori*, they are involved in cell shape maintenance, are essential for motility in *B. subtilis*, and a relatively large number of bactofilins play an important role in chromosome segregation and also cell shape maintenance in *Myxococcus xanthus* (Sichel and Salama, 2020). Cap0037 affects its own transcript levels, and thereby also those of the *C. acetobotylicum* bactofilin, but the functional relevance of this effect is unclear. *C. crescentus* bactofilins interact with enzymes involved in cell wall synthesis (Kuhn et al., 2010), which is also the case for *H. pylori* CcmA (Holtrup et al., 2022; Sichel et al., 2022). How these interactions affect the architecture of the cell wall is still unclear, but a connection of bactofilins to integral membrane proteins (Holtrup et al., 2022; Sichel et al., 2022) is a recurring theme.

**Figure 1:**
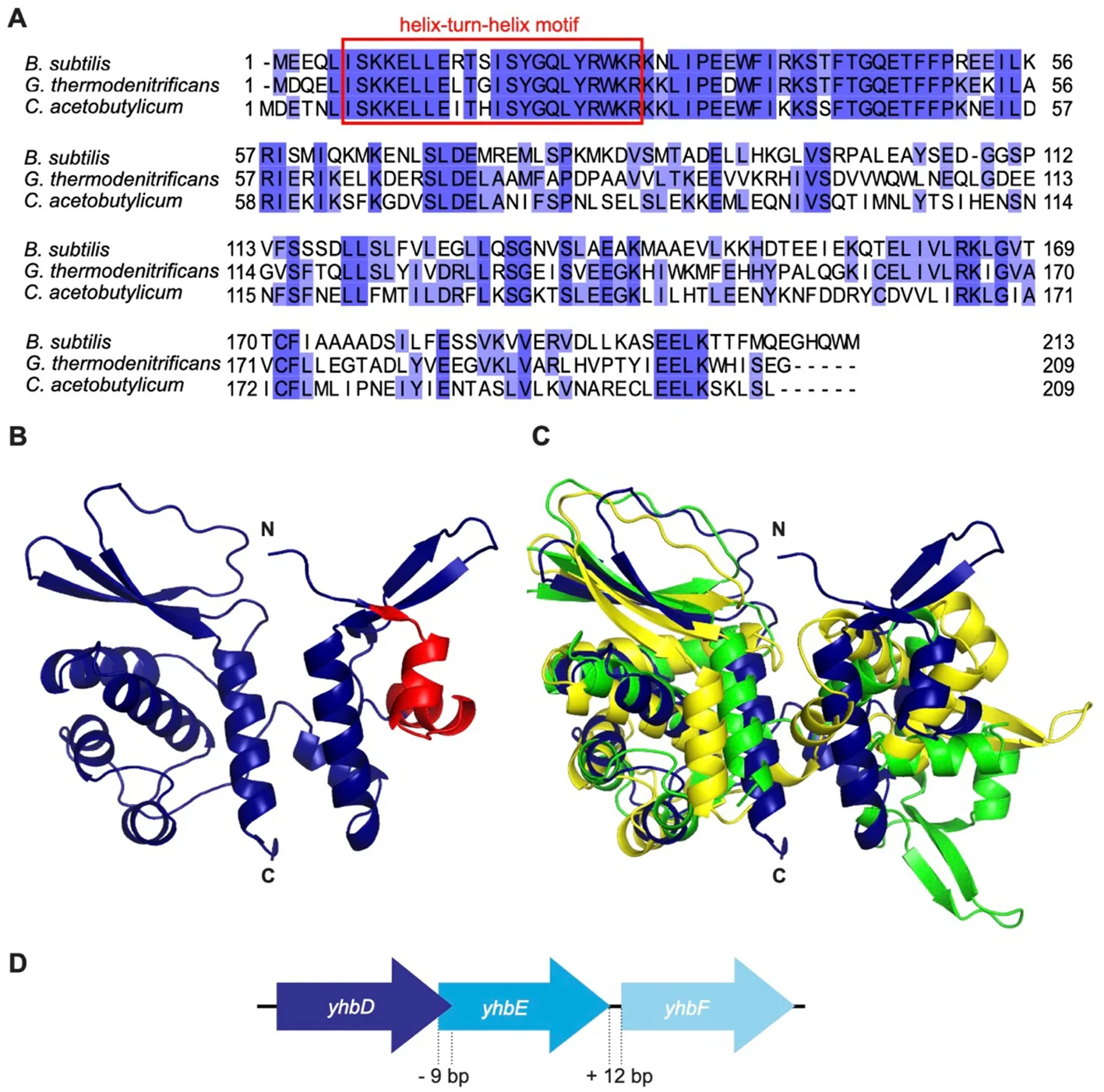
*In silico* characterization of BacR. **(A)** Amino acid sequence alignment of the proteins BacR from *B. subtilis*, from *G. thermodenitrificans*, and Cap0037 from *C. acetobutylicum*. The predicted Helix-turn-helix motif of BacR is highlighted by the red box. The alignment was generated by Clustal Omega (Madeira et al., 2024) and visualized by Jalview 2.0 (Waterhouse et al., 2009). **(B)** Predicted structure of *Bs*BacR protein (P97032, blue) by AlphaFold 3.0 (Abramson et al., 2024). The predicted Helix-turn-helix motif is shown in red. **(C)** Structural alignment of AlphaFold 3.0 predicted structures of BsBacR (P97032, blue), GtBacR (A4IPT8, green), and CaCap0037 (Q97TR1, yellow). N: (N-terminus), C: (C-terminus). **(D)** Operon structure of gene *yhbD* and the bactofilin genes *yhbE* (BacE) and *yhbF* (BacF) in *B. subtilis*.

We show that opposed to global gene regulation like its Clostridial counterpart, YhbD from *B. subtilis* affects mRNA levels of a single operon, containing its own gene and those of the bactofilins. We therefore propose to rename YhbD as “BacR” for regulator of bactofilins. BacR is part of a switch-like mechanism involving membrane-localized bactofilins, depletion of which allows BacR to localize to the nucleoids. Such a switch visibly occurs when cells are treated with vancomycin, indicating that BacR may be involved in signal transduction during cell wall stress.

## 2 Results

### 2.1 *B. subtilis yhbD* encodes for a putative transcription factor (BacR) that is widely conserved in Firmicutes

*YhbD* encodes for a protein that is a member of the DUF4004 (pfam 13171) protein family. Homologs of the *yhbD* gene product are conserved in many species of *Bacilli* and *Clostridia* (Supplementary Figure S1B), of the proteins identified, 74% are found in members of the *Bacilli*, 25% in *Clostridia*, and 1% was detected in Archaea, specifically in *Methanobacterium*. The latter may be a case of horizontal gene transfer. The most highly conserved homologs occur within the order *Bacillales*, including many species of the genera *Paenibacillus, Bacillus, Neobacillus*, and *Parageobacillus* (*Geobacillus*; Supplementary Figure S1B). The protein family currently includes 972 members, all approximately 210 amino acids in length (Blum et al., 2025). Many proteins within this family are predicted to be cytoplasmic, including YhbD from *Bacillus licheniformis* (58.4% sequence identity) and BGAA_2880 from *Bacillus anthracis* (51.2% sequence identity) (Galperin et al., 2021). Indeed, no transmembrane domain can be identified for BacR from *B. subtilis* using DeepTMHMM (https://dtu.biolib.com/DeepTMHMM/). Additionally, many of the DUF4004 proteins possess a conserved N-terminal helix-turn-helix (HTH) motif, suggesting a potential DNA-binding capacity (Figure 1B). For BacR, the predicted HTH motif consists of the sequence ISKKELLERTSUSYGQLYRWKR. The C-terminal region of BacR and of other DUF4004-containing proteins exhibits overall low sequence identity (Figure 1A).

A comparison of BacR with Cap0037 from *C. acetobutylicum* (44.3% sequence identity) and BacR from *Geobacillus thermodenitrificans* highlights the similarity of these proteins (Figure 1A), particularly in their N-terminal regions. Of note, the start codon of *yhbD* in the Subtiwiki database (http://subtiwiki.uni-goettingen.de) is most likely a mis-annotation, because (a) there is a transcriptional upshift after the first annotated ATG, and (b) BacR homologs from other bacteria do not contain a sequence upstream the HTH motif (Figure 1A). AlphaFold-based structural comparison (Figure 1C) reveals strong structural conservation in the C-terminal region, despite low sequence identity, and more divergence in the N-terminal structure, in spite of higher sequence similarity [for confidence metrics and Root Mean Square Deviation (RMSD) values see Supplementary Figure S2]. Sequence and structural similarities support the hypothesis that BacR may function as a transcriptional regulator. As will become apparent below, the *yhbD* gene product indeed affects transcription of the *yhbDEF* operon, wherefore we propose to name it “BacR” as “bactofilin regulator”.

The presence of DUF4004s in diverse taxonomic groups indicates a potentially important and evolutionarily conserved role that extends beyond the genus *Bacillus*. DUF4004 proteins are also present in bacteria inhabiting extremophilic environments, such as *Parageobacillus thermoglucosidasius* and *Geobacillus thermodenitrificans* (Supplementary Figure S1B). The *yhbD* gene is first of a three-gene operon in *B. subtilis* (Figure 1D). Supplementary Figure S1A shows that in several species, the gene is upstream of one or two genes encoding for bactofilins.

### 2.2 BacR affects mRNA levels of the *yhbDEF* operon

We employed RNAseq analyses to determine if BacR might exert a function as TF in *B. subtilis*, similar to its homolog in Clostridium. As reference, we treated cells with the cell wall antibiotic vancomycin, as the response is well documented (Radeck et al., 2017). Wild type and in-frame *yhbD* mutant cells were grown exponentially, or were stressed with vancomycin for 30 min, and transcript levels were determined using RNA sequencing (see Supplementary Figure S3 for quality controls). In response to vancomycin (4 μg/mL), we observed significant up- and downregulation of many genes (725 upregulated, 845 downregulated; padj < 0.1, |log2FC| > 1; Figure 2A, Supplementary Figure S3A), which were previously characterized as components of the Cell Envelope Stress Response (CESR) (Radeck et al., 2017). Figure 2A is a combined analysis; upper left quadrant shows genes upregulated in mutant cells and downregulated in wild type cells, lower right quadrant genes upregulated in wild type and down regulated in mutant cells, other quadrants genes up or downregulated in a similar manner in both strains; all spots are gray because no significant changes were found. Figure 2B shows selected results from a triplicate analysis, illustrating upregulation of several CESR operons and downregulation of *tasA*, as reported earlier. These data validate the quality of our transcriptome analyses. Of note, the *yhbDEF* operon did not show considerable changes in transcript levels in response to vancomycin, neither in wild-type cells nor in Δ*yhbD* mutant cells (Figure 2B). The minor fluctuations observed in *yhbDEF* transcript levels in response to vancomycin are attributable to the generally low abundance of these transcripts, and do not reach statistical significance.

**Figure 2:**
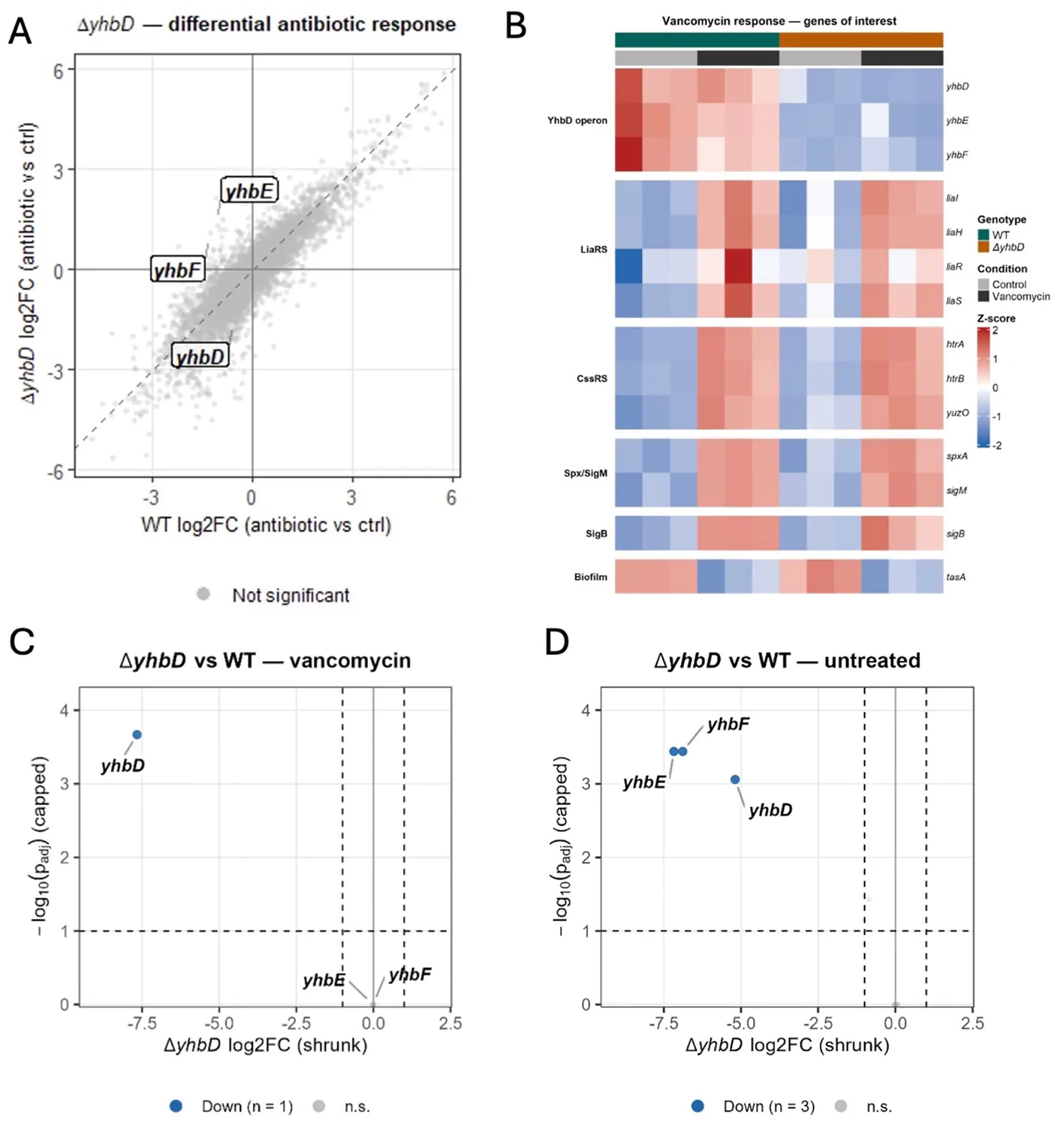
RNAseq analysis of *B. subtilis* wild type or *yhbD* mutant cells, growing exponentially (Ctrl) or 30 min after treatment with vancomycin Van0. **(A)** Scatter plot showing differences between exponentially growing cells and cells treated with vancomycin. **(B)** Heat map of changes seen for selected genes, in triplicate. **(C)** Logarithmic volcano plot of differences seen between wild type and *yhbD* mutant cells in response to vancomycin. *YhbE* and *yhbF* are shown as examples for all genes, whose differences are not significant, and therefore lie closely around “0”. **(D)** Logarothmic volcano plot of differences seen in exponentially growing wild type and *yhbD* mutant cells.

Likewise, overall, we did not detect significant differences between the global CESR in wild-type and Δ*yhbD* mutant cells (Figure 2C, Supplementary Figures S4A, B). Formal testing of the genotype-treatment interaction yielded no differentially expressed genes, demonstrating that the loss of BacR does not measurably affect the transcriptional stress response to vancomycin. The sole detectable difference was found under non-stress conditions, where transcript levels of *yhbD, yhbE*, and *yhbF* were significantly lower in mutant cells than in wild-type cells (log2FC = −5.2, −7.2, −6.9 respectively; padj < 0.001; Figure 2D). Thus, BacR has a positive effect on the transcription of its own operon, be it directly as a transcriptional regulator, or indirectly.

### 2.3 BacR is a DNA-binding protein

For a biochemical charactarisation of BacR and for testing, if it may excert its positive effect on transcription directly via binding to DNA, we purified BacR from *B. subtilis* and from the related, thermophilic organism *Geobacillus thermodenitrificans*. It was impossible to obtain the proteins in a soluble manner in sufficient amounts. Solubility was improved using an N-terminal glutathione-S-transferase (GST) fusion to BacR (Figures 3A and B, Supplementary Figure S5). Although we performed all analyses for both proteins, we will concentrate here on results obtained for BacR protein from *G. thermodenitrificans* (GtBacR, 47.8% sequence identity to BsBacR), for which we obtained more reproducible larger quantities. The conserved N-terminal HTH-motif differs by only two amino acids from that of BsBacR (R39L, S41G; Figure 1A). The proteins exhibit high predicted structural similarities (Figure 1C). GtBacR was heterologously expressed in *E. coli* with an N-terminal StrepII-tag and was purified via streptavidin affinity chromatography (Figure S5). The eluted protein migrated at a molecular weight of approximately 29 kDa, corresponding to the expected size of Strep-GtBacR (28.8 kDa). The protein was successfully isolated from *E. coli* cells (Figure 3A, lanes 7–10), high purity was achieved after concentrating the eluted protein (Figure 3B, lane 1). To determine the oligomeric state of the protein, analytical size exclusion chromatography (SEC) was performed using the concentrated protein (Figure 3B, lane 1). The chromatogram revealed a single peak corresponding to proteins with a molecular weight between 44 and 158 kDa (Figure 3C; peak fractions shown in Figure 3B, lanes 3–6). Because monomeric Strep-GtBacR has a molecular weight of 28.8 kDa, these results suggest that Strep-GtBacR exists in a higher-order oligomeric state. Low absorbance at 255 nm indicates negligible nucleic acid contamination. To more precisely determine the oligomeric state of Strep-GtBacR, mass photometry was performed. The analysis revealed a single distinct peak corresponding to a molecular weight of 50 ± 7.6 kDa (Figure 3D, blue), suggesting that the protein predominantly exists in a dimeric form *in vitro* (calculated dimer: 57.6 kDa). Notably, 97% of the detected counts matched this molecular weight, indicating a highly homogeneous oligomeric state. A protein standard measured in the same buffer was used for calibration to ensure the accuracy of mass determination (Figure 3D, gray).

**Figure 3:**
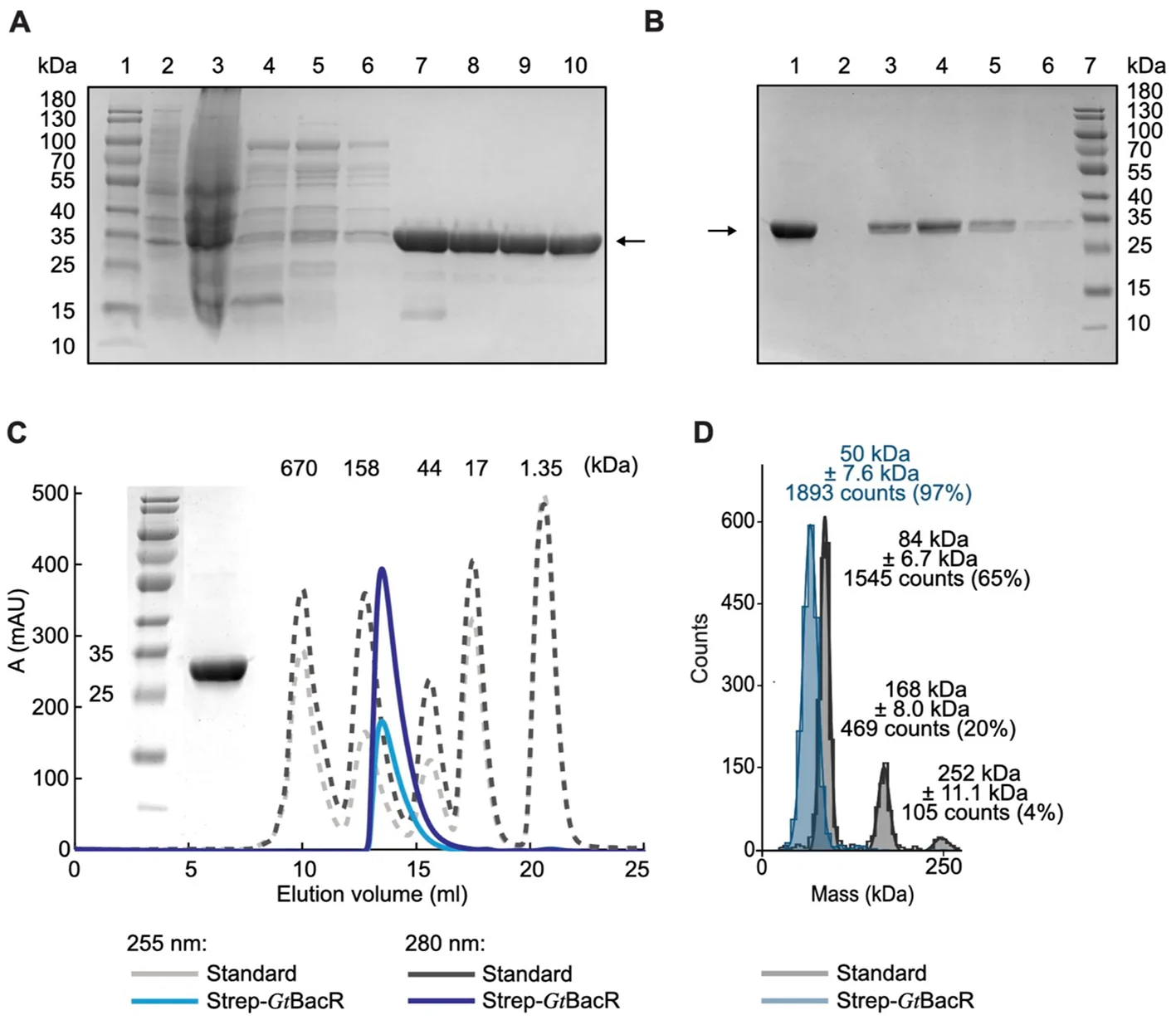
Purification and characterization of Strep-GtBacR. (A) Coomassie-stained SDS gel of purification steps of Strep-GtBacR (~ 29 kDa) by streptavidin chromatography. Lane (1) represents pre-stained protein marker, (2) cells, (3) lysed cells, (4) lysate, (5) flow through, (6) wash and (7–10) elution fractions. **(B)** Respective Coomassie-stained SDS gel with lane (1) concentrated protein loaded on SEC and mass photometry (2) nothing applied, 3–6 samples represent the peak of SEC and (7) represents pre-stained PageRuler. **(C)** Chromatogram of analytical size exclusion chromatography of concentrated Strep-GtBacR. The light blue line displays an absorbance at 255 nm, and the dark blue line displays 280 nm. The standard is shown in a dashed gray line. **(D)** Mass Photometry of purified concentrated Strep-GtBacR. The standard is labeled gray, and Strep-GtBacR is blue.

Further evidence for a potential dimerization region was obtained through AlphaFold 3.0 prediction. The models suggest that BacR monomers may approach each other via a C-terminal structure, potentially contributing to dimer formation. This interaction in the C-terminal region was observed for both BsBacR (Supplementary Figure S6A) and GtBacR (Supplementary Figure S6B). An alignment of the predicted structures further supported the hypothesis of similar dimerization behavior, as both proteins exhibited highly similar structural features in this region (Supplementary Figure S6C).

To test for a potential autoregulatory function of BacR, it was analyzed whether GtBacR, like Cap0037, can bind to the promoter region of the *yhbD-bacEF* operon. As the precise position of the operon's promoter region is unknown, the BPROM tool (https://sapphire.biw.kuleuven.be) was used to predict potential promoter sequences. Four possible promoter regions were identified: one in the upstream gene *cspR*, two within the *yhbD* gene, and one in *bacE* gene. Electromobility shift assays (EMSA) of the promoter region predicted upstream of the *yhbD* gene showed clear shift of the double-stranded DNA (dsDNA, 42 bp; Figure 4A). A clear shifted band was present at the lowest protein concentration, indicating that a specifically recognized sequence may be within this region. GtBacR also bound to 646 bp and to 1,636 bp fragments covering all identified promoter sequences (Figures 4B and C). Interestingly, binding efficiency increased with the length of the DNA, as progressively less unshifted DNA was detected at the lowest protein concentrations, with increasing fragment size (Figures 4A–C). This effect hampered our efforts to identify (short) sequences of preferred BacR binding. EMSAs using single-stranded DNA (ssDNA) revealed only a minor fraction of shifted DNA at increasing concentrations of Strep-GtBacR (Figure 4D), showing pronounced preference for dsDNA. Further analyses regarding DNA-binding specificity showed that GtBacR can also bind to DNA fragments from unrelated genes, such as *hag* from *B. subtilis* or *murF* from *H. pylori* (Figures 4E and F). Binding efficiencies were lower but in a similar range as for the DNA region of *yhbD-bacEF* operon.

**Figure 4:**
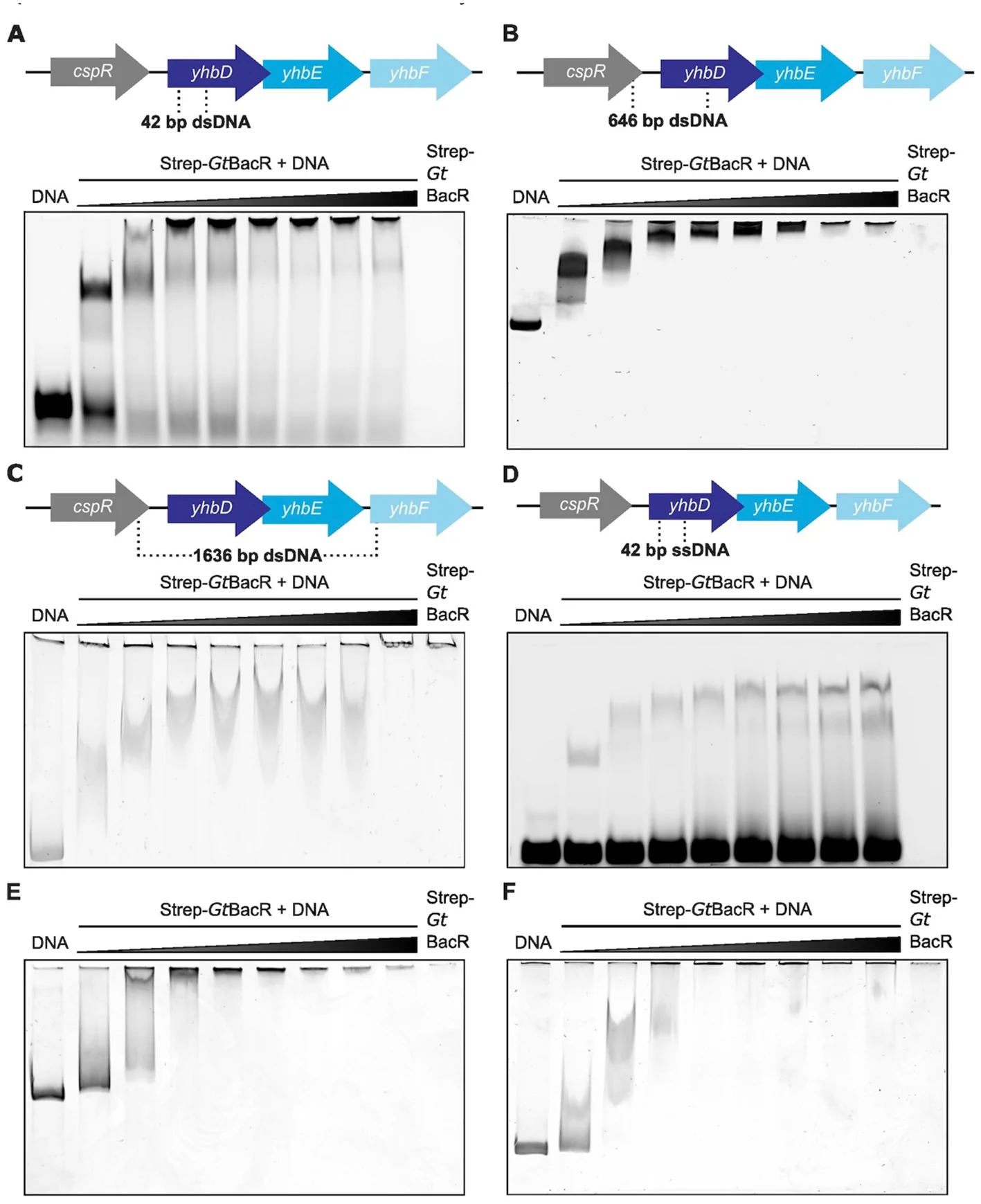
DNA binding of Strep-GtBacR. Midori Green stained native PAGE of electromobility shift assays using Strep-GtBacR (2–16 μM) and different DNA fragments. **(A–D)** Parts of the *yhbDEF* operon; **(A)** 42 bp dsDNA of predicted **–**30 **–**10 promoter region, **(B)** 646 bp dsDNA, **(C)** 1,636 bp dsDNA, **(D)** 42 bp ssDNA of predicted **–**30 **–**10 promoter region. **(E)** EMSA with a part of *hag* gene of *B. subtilis* (1,700 bp)and **(F)** a part of *murF* gene of *H. pylori* (1,460 bp).

We tested the impact of divalent ions on DNA binding, and found that zinc ions had a positive effect in DNA binding of BsBacR, but no such effect was observed for magnesium, manganese or copper (Supplementary Figure S7). Because zinc increases DNA-binding affinity for many transcription factors other than zinc-finger proteins, this effect on BacR could be non-specific, but is nevertheless noteworthy.

### 2.4 Bactofilins GtBacE and GtBacF interact with GtBacR and interfere with its DNA binding activity

The genetic context of the *yhbD-bacEF* operon prompted us to investigate a possible interaction between the proteins. Interaction models of BacR with BacE and BacF were generated using AlphaFold 3.0 (Figures 5A, B, GtBacR highlighted in green, BacE in dark blue and BacF in cyan) suggest a potential interaction interface between BacR and the bactofilins is near the BacR helix-turn-helix motif (Figures 5A, B, red, Supplementary Figure S2).

**Figure 5:**
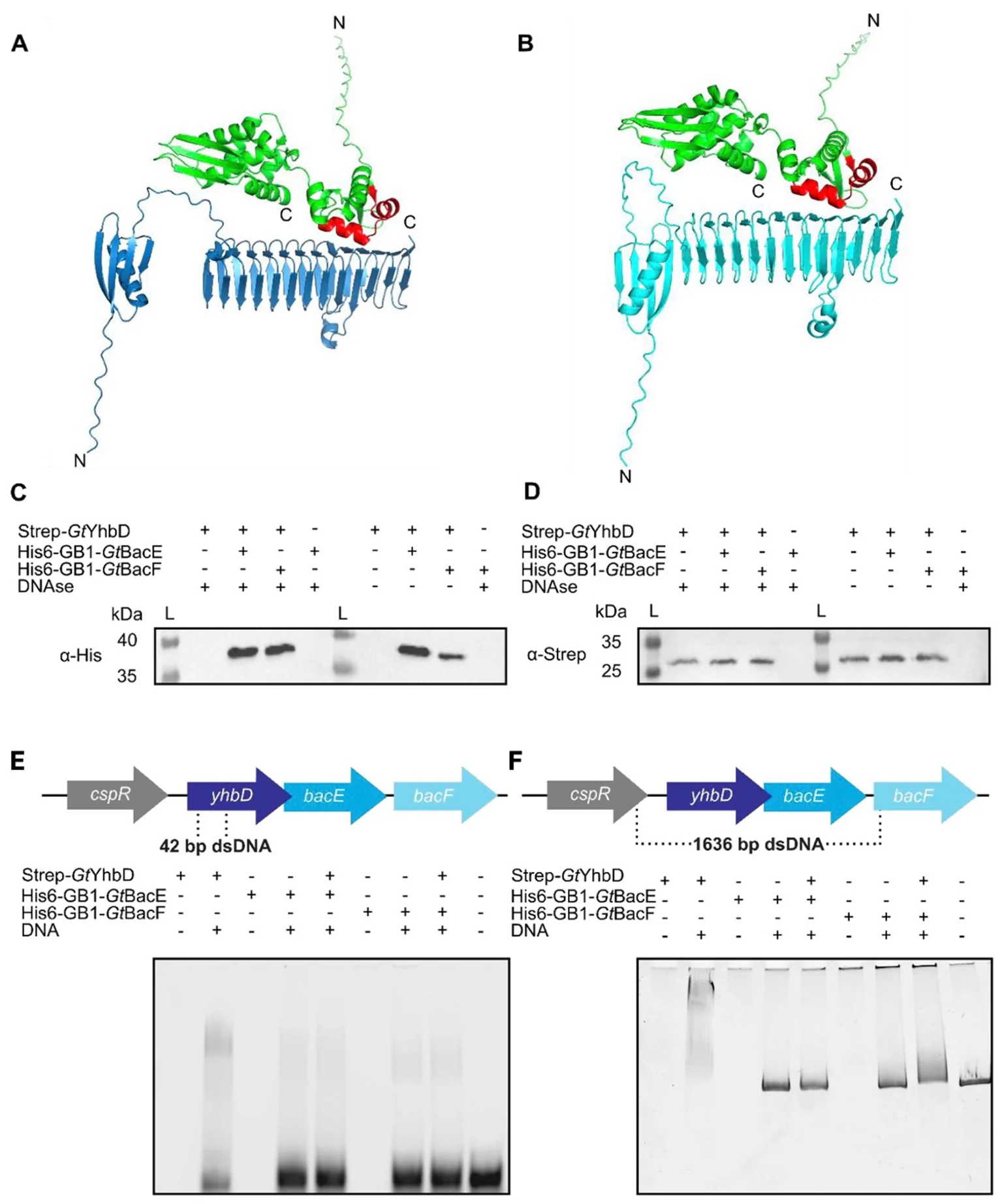
Interaction study of GtBacR and the bactofilins GtBacE and GtBacF. **(A, B)** AlphaFold3 model of an *in silico* predicted interaction between Strep-GtBacR monomer (green) and His6-GB1-GtBacE monomer (A, dark blue) or His6-GB1-GtBacF (B, cyan blue). The helix-turn-helix motif of Strep-GtBacR is shown in red. “N” and “C” indicate the N-terminus and C-terminus of the respective proteins. **(C, D)** Western blot analysis of an *in vitro* pulldown assay with Strep-GtBacR (28.8 kDa) as bait protein and His6-GB1-GtBacE (34.3 kDa) or His6-GB1-GtBacF (35.5 kDa) as prey protein by streptavidin beads. His-tagged proteins were detected with an α-His antibody **(C)** and Strep-tagged proteins were detected with α-Strep antibody **(D)**. Lane L represents pre-stained. **(E, F)** DNA binding of Strep-GtBacR in the presence and absence of the bactofilins GtBacE and GtBacF. Midoori Green-stained native PAGE of EMSAs of Strep-GtBacR with different DNA regions in the presence and absence of the bactofilins His6-GB1-GtBacE and His6-GB1-GtBacF. **(E)** 42 bp dsDNA of the predicted **–**30/ **–**10 promoter region of the *yhbD-bacEF* operon. **(F)** 1,636 bp dsDNA of *yhbD-bacEF* operon.

To test these predicted interactions *in vitro*, purified GtBacR and bactofilins from *G. thermodenitrificans* were used (same operon structure as in *B. subtilis*). StrepII-tagged GtBacR and His6-GB1-tagged GtBacE or GtBacF was recombinantly produced in *E. coli* BL21 C41 (DE3). Lysates from cells producing Strep-GtBacR were combined with purified bactofilin proteins, and the mixtures were applied to Strep-Tactin beads for pull-down assay. As controls, lysates of the individual proteins were applied to the column separately (Figures 5C, D, lanes 1, 4, and 8). The results demonstrate that GtBacE and GtBacF co-eluted with GtBacR (Figures 5C, D, lanes 6 and 7), supporting a direct physical interaction between BacR and the bactofilins.

Additionally, we investigated whether the presence or absence of bacofilins affects DNA-binding of BacR (Figure 4), in light of the AlphaFold 3.0 prediction that bactofilins interact with the HTH-motif of BacR (Figures 5A, B). For this purpose, pulldown assays were performed using lysates preincubated with or without DNase. Figures 5E, F show that while BacR binds to dsDNA on its own (second lanes), this capacity is strongly reduced/abolished in the presence of BacE or of BacF, especially for the long DNA fragment (Figure 5F). Thus, DNA and bactofilin-association appear to be mutually excluded for BacR, such that the capability of BacR to bind to DNA is strongly reduced in the presence of bactofilins.

### 2.5 BacR localizes to the cell membrane dependent on the presence of bactofilins

To further characterize BacR *in vivo*, its intracellular localization was investigated. We generated a *B. subtilis* strain in which BacR is fused with mNeonGreen (mNG) at its C-terminus. The fusion construct was integrated into the native gene locus via a single crossover event, placing the downstream operon genes *yhbE* and *yhbF* under the control of a xylose-inducible promoter. This promoter can be activated by adding xylose and is repressed in the absence of xylose by adding glucose (Figure 6A). This strategy enabled us to investigate the subcellular dynamics of BacR during continued or repressed expression of the downstream bactofilin genes *bacE* and *bacF*.

**Figure 6:**
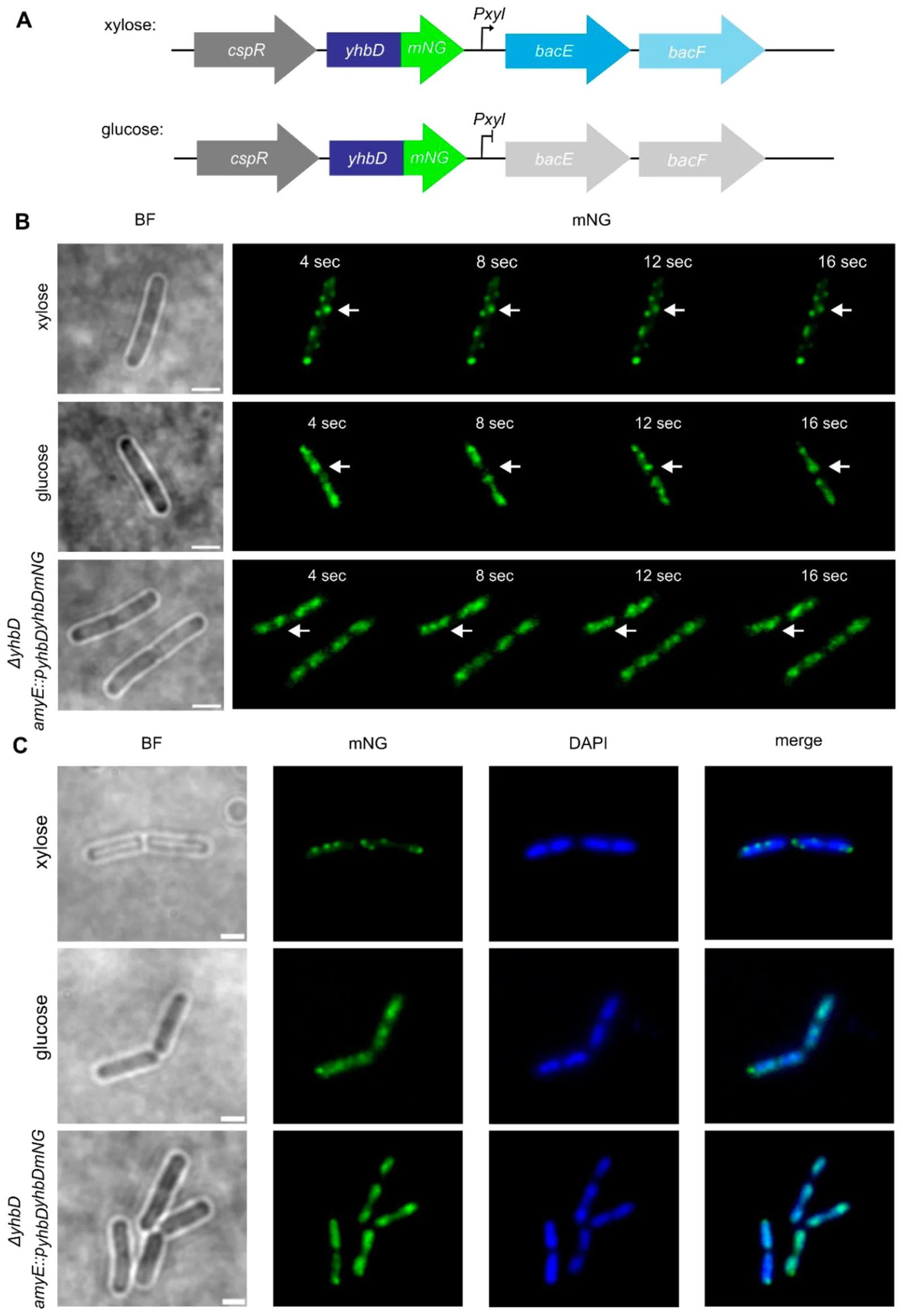
Fluorescence microscopy of the BacR-mNG fusion in 3610 DK1042. **(A)** Illustration of the integration of *yhbD-mNG* into the native locus through a single crossover. The downstream genes *yhbE/bacE* and *yhbF/bacF* are controlled by a xylose-inducible promoter that is repressed by glucose. **(B)** Images of time-lapse microscopy of BacR-mNG, four s intervals. Shown are cells with a *yhbD-mNG* fusion in the original locus treated with glucose or xylose (as described in A). In addition, cells were shown with ectopically expressed *yhbD-mNG* with the native promoter at the *amyE* locus in a *yhbD* deletion Relevant areas are marked with white arrows. **(C)** Epifluorescence microscopy of BacR-mNG as described in **(B)**. The chromosome was stained with DAPI. Merge shows the overlay of the BacR-mNG signal and the DAPI signal. Scale bars 2 μm.

In the presence of xylose BacR-mNG predominantly localized in foci at the cell membrane (Figure 6B), and thus largely away from the centrally localized nucleoids. Time-lapse microscopy revealed that these membrane-associated foci remained largely static over the observation period of 16 s (Figure 6B). Interestingly, in cells with repressed bactofilin expression BacR-mNG no longer formed membrane-associated foci but was detected in a pattern similar to that of the nucleoids (Figure 6B, middle panel). Time lapse experiments showed that the pattern of localization changes between four-second-intervals, in contrast to the static formation of membrane-associated foci in the presence of BacE and BacF. At different time point, BacR-mNG also shows discrete accumulations on the nucleoids; staining of DNA using DAPI (Figure 6C, middle panel) revealed that indeed, BacR does not uniformly localize on the nucleoids in the absence of bactofilins. These experiments suggest that in the absence of bactofilins, BacR moves between different sites on the nucleoids, possibly in search for specific binding sites.

A notable caveat in our analyses is the fact that we can not control the exact copy numbers of BacE and BacF, using the xylose promoter. Induction with xylose may lead to an overproduction of bactofilins, which may exaggerate the formation of membrane foci. We can clearly state that the repression of bactofilins abolishes membrane localization, but the true degree of membrane-association may be less pronounced than what is visible in the micrographs in Figure 6.

To analyze whether the genomic proximity of *yhbD* to the bactofilin genes is functionally relevant, 250 bp upstream of the *yhbD* gene, including its putative promoter, were cloned together with *yhbD-mNG* into the *amyE* locus in an in-frame *yhbD* deletion strain (3,610 Δ*yhbD amyE::PyhbD-yhbD-mNG*). This construct enabled the native expression of the bactofilins from their original genomic locus, whereas *yhbD-mNG* was expressed at a different genomic location. Interestingly, the pattern of BacR-mNG localization resembled that of cells with repressed bactofilin expression (Figures 6B and C, lower panels). Only, few membrane-associated foci were observed, while most of the signal was dynamically distributed at the nucleoid region. Thus, membrane-association of BacR depends on co-transcription with bactofilin genes.

Epifluorescence microscopy integrates fluorescent signals over several hundred milliseconds, largely missing molecules that freely diffuse through the cell. To obtain more detailed information on the mobility of BacR-mNG, single molecule tracking was performed. Trajectories were analyzed using squared displacement analyses, and visualized by jump distance (JD) plots (Oviedo-Bocanegra et al., 2021). Non-linear regression (Rayleigh fitting) was used to model the data. Although using two Rayleigh fits sufficiently represent the data (Supplementary Figure S8A, top), we used three Rayleigh fits, which were required to fully cover all observed Pdfs (Supplementary Figure S8B, Figure 7). Given that BacR binds to dsDNA, we assumed the existence of a freely diffusive population, a population of molecules showing non-specific on—and off rates of binding on the nucleoids, and a static population that is either tightly bound to DNA, or present in the observed static foci at the cell membrane (Figure 6). Based on the JD data, the fastest population (Pop_fast − mobile_) comprised approximately 28% of all BacR molecules under normal conditions, with a diffusion coefficient (D_fast − mobile_) of 0.66 μm^2^/s (Figure 7B). The slow-mobile and most abundant population (Pop_slow − mobile_) accounted for around 47% of BacR molecules, with a diffusion coefficient (D_slow − mobile_) of 0.14 μm^2^/s. The slowest population (Pop_static_) represented about 25% of the molecules, with a diffusion coefficient (D_static_) of 0.03 μm^2^/s (Figure 7B, green). During the repression of bactofilin expression, the freely diffusive population (Pop_fast − mobile_) did not change, while the slow-mobile population (Pop_slow − mobile_) moderately increased, at the expense of the static population (Pop_static_; Figure 7B). These results show that relocation of BacR-mNG to the nucleoids is not accompanied by large changes in the distribution of mobilities of BacR-mNG populations in the cell.

**Figure 7:**
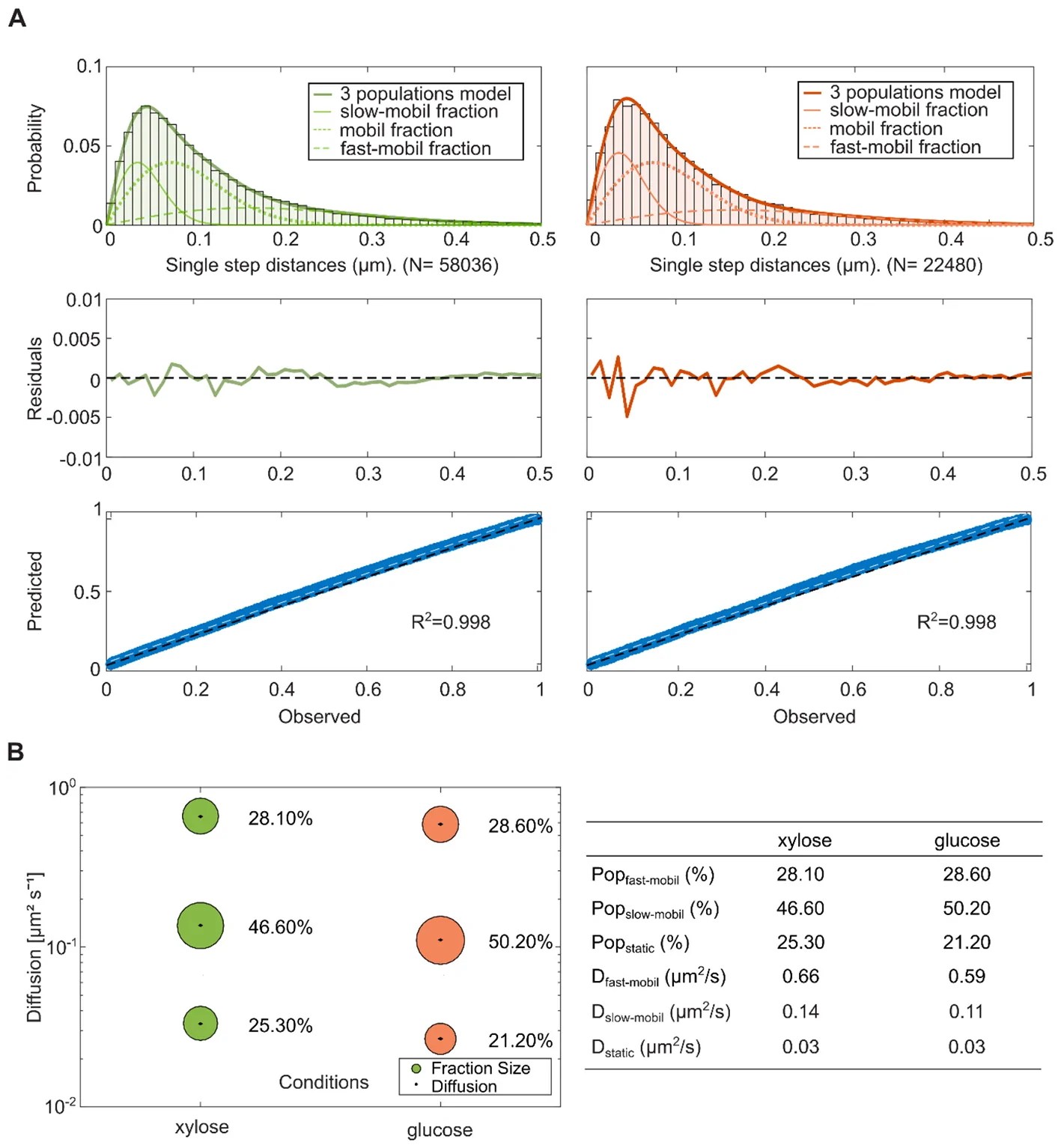
Squared displacement analysis (SQD) of BacR-mNG in cells treated with glucose or xylose in 3610 DK1042. (**A**) Three Rayleigh distributions were used to fit the observed data and calculate diffusion coefficients for individual populations (N: Number of trajectories generated by triplicates). The frequency of the diffusion constants (probability density) is shown as a function of the diffusion coefficient in a histogram. The respective coefficient of determination (*R*^2^) shows quality of the fits. Lower panels display corresponding quantile-quantile plots, which illustrate the comparison between the measured (dashed line) and modeled (blue line) distributions of the three populations. **(B)** Bubble plots show diffusion coefficients (μm^2^/s) and the relative proportions of the populations (%) of BacR-mNG fusions. Table shows diffusion constants and percentages of fraction sizes calculated by a nonlinear least-squared fitting method.

To assess the localization of BacR-mNG molecules using single molecule tracking (SMT), observed trajectories were projected onto a normalized *B. subtilis* cell of average size (3 × 1 μm; Figure 8). Heat maps of molecules exhibiting confined motion, which was defined as movement within a spatially limited region of no more than 106 nm in diameter, showed a clear redistribution of molecules from the membrane under normal conditions onto the nucleoids during bactofilin depletion (Figure 8A). When all tracks were used for the generation of heat maps, changes also closely matched those observed via epifluorescence microscopy, a large change of the molecules from the cell periphery toward the cell center upon repression of bactofilin expression (Figure 8B). Heat maps show the presence of tracks away from the cell membrane under normal conditions (i.e., freely diffusive molecules), and throughout the nucleoids and less pronounced at the cell poles under glucose conditions. These analyses show that more than 70% of all BacR molecules show motion associated with binding to large protein complexes or to the chromosome, and a rather small free population.

**Figure 8:**
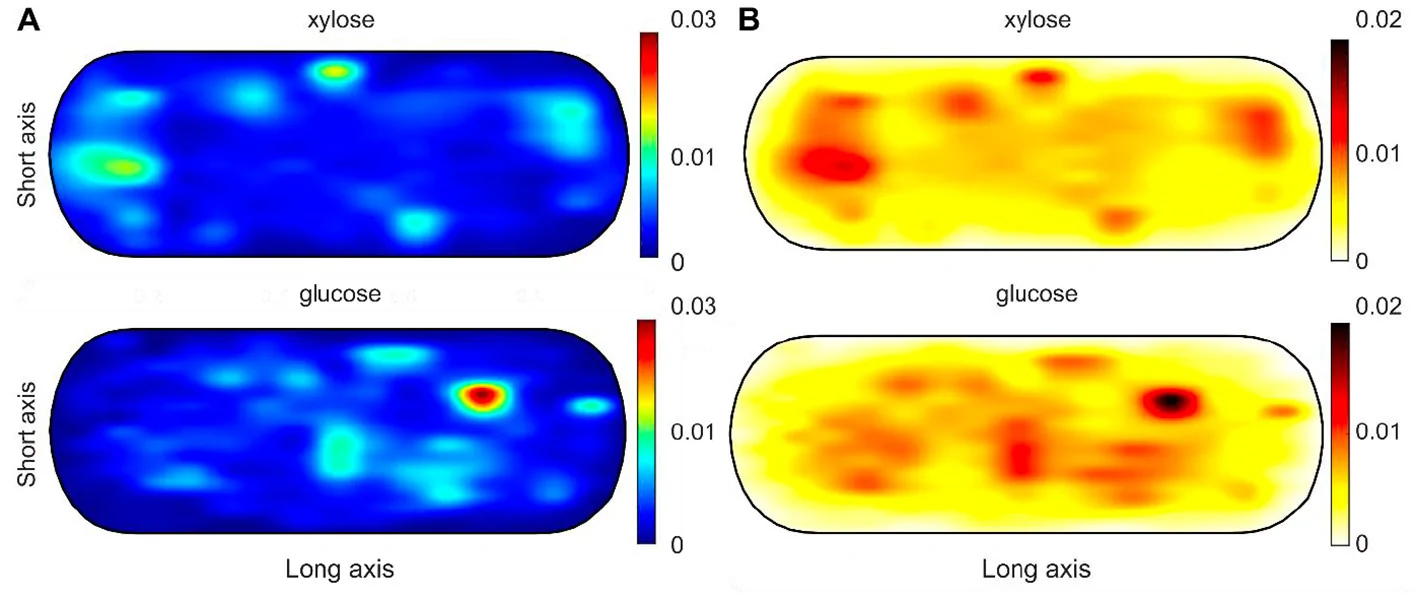
Localization of BacR-mNG. 3610 DK1042 *B. subtilis* cells with BacR-mNG fusion were grown by adding xylose or glucose during induction or repression of the expression of bactofilins. The *yhbD-mNG* strain expresses this allele from its native locus. **(A)** Heat maps of confined tracks of BacR-mNG fusion. The color codes on the right indicate the probability of the molecule's presence. Low (blue) and high (red). The cells represent a standardized (average size) cell with a width of 1 μm and a length of 3 μm. **(B)** Heat maps of the localization of all single molecules of BacR-mNG fusion. Color codes indicate the probability of the molecule's presence. Low (yellow) and high (red). The cells represent a standardized cell with a width of 1 μm and a length of 3 μm.

### 2.6 Cell fractionation reveals peripheral membrane association of BacR and an influence of bactofilins on BacR levels

The strong visual association of BacR molecules to the cell membrane prompted us to study membrane association with biochemical means. Cell fractionation followed by Western blot analysis was performed using exponentially growing cells. As a control for integral membrane proteins, SecDF-mNG was employed. This 100 kDa protein was exclusively found in the membrane fraction (Figure 9D), whereas cytosolic protein DNA polymerase PolA-mNG (125.8 kDa) was detected in the cytosolic fraction only (Figure 9D). In agreement with *in vivo* analyses above, BacR was detected in both membrane and cytosolic fractions (Figure 9A), revealing that it is associated with the membrane, bearing in mind that bactofilins may be overproduced using the xylose-inducible system. The fractionation shown in Figure 9A contains membrane–and cytosolic fractions that–taken together–correspond to the total lysate. This indicates that BacR is largely present in the cytosolic fraction, with the caveat that some molecules may detach from the membrane due to the fractionation procedure. Unlike PolA (Figure 9D), a considerable amount of BacR was found in the membrane fraction, suggesting that the observed foci are due to a direct membrane association or interaction. Different from SecDF (Figure 9D), BacR does not appear to be integrated into the membrane, suggesting that BacR association with the membrane is indirect, indicating that the protein can switch between membrane-associated and soluble mode. Interestingly, Western blot analysis revealed bands specific for BacR-mNG lower than full-length protein, present in the cytosolic, but not in the membrane fraction (Figures 9A, C and F). This observation strongly suggests that BacR is prone to proteolysis when soluble within the cytosol. Note that the lowest degradation band should correspond to free mNeonGreen (~27 kDa; Supplementary Figure S9A), suggesting that some of the cytosolic signal observed in microscopy stems from free mNeonGreen. However, due to imaging conditions using 20 ms integration time, most of the free mNeongreen signal is not captured during tracking, because a much higher imaging speed is required to capture all tracks of a 27 kDa protein (Schibany et al., 2018).

**Figure 9:**
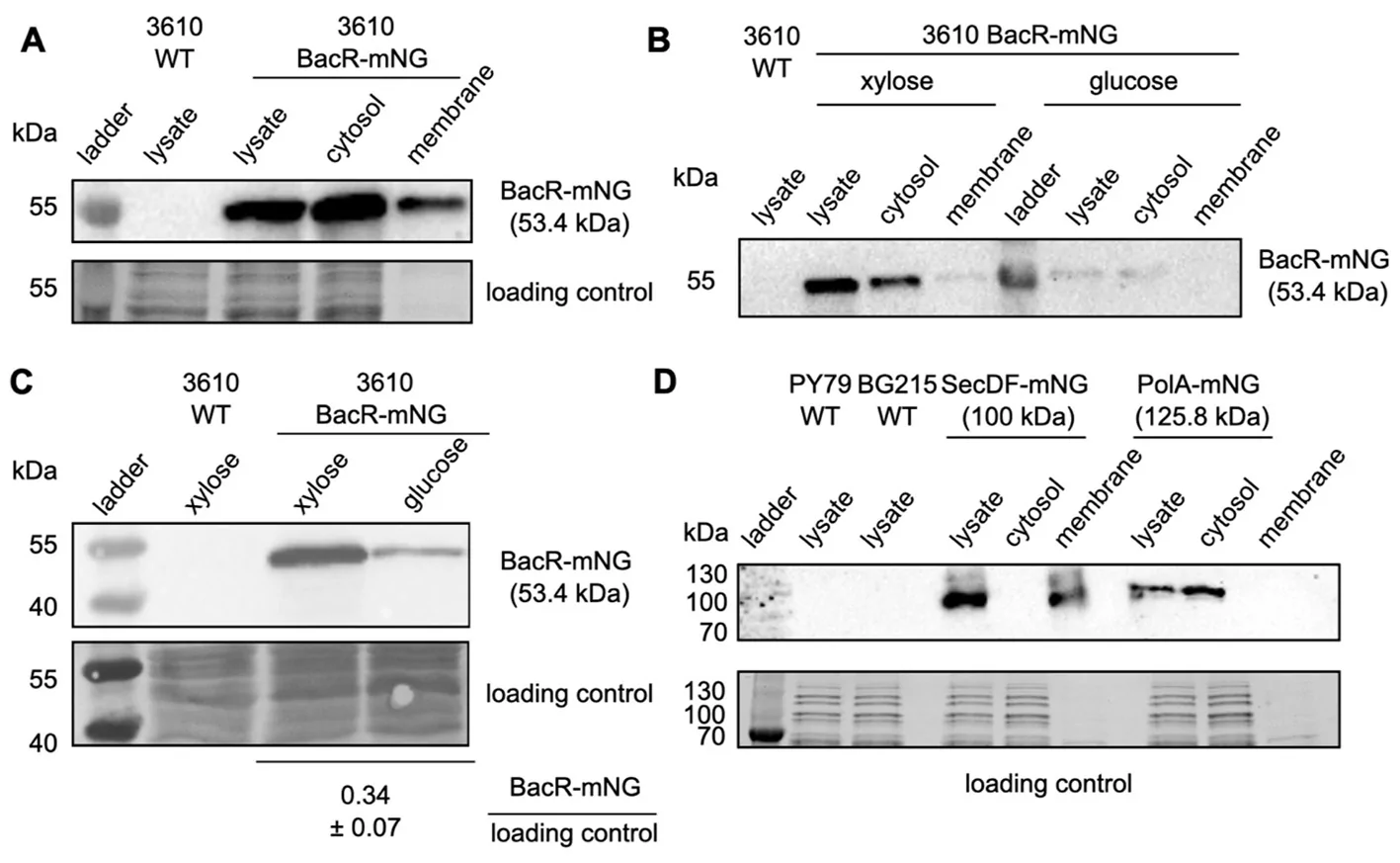
Cell fractionation and quantification of BacR-mNG. **(A–D)** Western blot analysis of protein-mNG fusions using anti-mNeongreen antiserum. Amido black staining was used for loading controls (lower panels). **(A, B, D):** fractions were loaded such that the amount of protein from cytosol and membrane add up to the amount of protein in the lysate, whereas in **(C)**, the same amount of protein was loaded to quantify BacR levels between conditions of bactofilin expression (xylose) or repression (glucose); number below the lanes indicates the mean level of BacR-mNG in cells grown with glucose relative to xylose condition; SD (±) is calculated from triplicates. **(A–C)** extracts from cells expressing BacR-mNG, **(A)** cells grown under bactofilin expression (xylose), **(B, C)** cells grown under bactofilin expression (xylose) or repression (glucose) conditions. **(D)** cell extract from cells expressing SecDF-mNG or PolA-mNG. Strain 3610 DK1042 (wild type cells without any mNG fusion) was used as control.

Given that the subcellular localization of BacR-mNG changed upon activation and repression of bactofilin expression, we further analyzed whether this shift can also be detected in cell fractionation experiments. Under glucose conditions, where bactofilins are absent, BacR was no longer detectable in the membrane fraction (Figure 9B). Notably, the overall BacR-mNG signal intensity was lower compared to xylose conditions. Indeed, cellular BacR levels were strongly reduced (to one-third) when the expression of bactofilins was repressed using glucose-containing medium (Figure 9C). This indicates that BacR expression is substantially reduced under the repression of bactofilin expression, suggesting that bactofilins may influence BacR protein levels. This agrees with the observation that in SMT experiments, 107 trajectories per cell were obtained under xylose conditions, but only 18 trajectories per cell under glucose-induced repression.

### 2.7 Vancomycin induces a switch in the localization of BacR

Following the observation that BacR-mNG exhibits bactofilin-dependent membrane-association (Figure 6), resembling the localization pattern of the bactofilins themselves (El Andari et al., 2015), and given the *in vitro* interaction between GtBacR and bactofilins (Figure 5), we investigated if conditions of membrane stress might induce a switch of BacR localization to the nucleoids, where BacR might become active in transcriptional regulation. For the following experiments, it must be kept in mind that the degree of the formation of membrane foci vs. localization to the nucleoids may be artificially shifted toward a formation of foci, due to the use of the xylose promoter for *yhbEF* (*bacE/bacF*) transcription. Thus, the experiments described below are to be interpreted regarding the relative changes in localization, which are truely reflected, rather than abolute numbers of localization patterns.

We tested various treatments causing stress to *B. subtilis* cells, including salt—and ethonol stress, which did not lead to any visible change in BacR-mNG localization (Supplementary Figure S10). We detected a striking response to vancomycin, a glycopeptide antibiotic that interferes with bacterial cell wall biosynthesis. Vancomycin binds to the D-Ala-D-Ala terminal residues of peptidoglycan precursors, thereby blocking their cross-linking and polymerization (Stogios and Savchenko, 2020).

Epifluorescence microscopy revealed that without treatment (w/o), BacR-mNG localized as membrane-associated foci in most cells, while vancomycin treatment led to a loss of membrane foci in most cells (Figure 10, Figure). We grouped cells into four categories, showing membrane foci only, a mixture between foci and cytosolic signal, cytosolic fluorescence-only, and DAPI-like staining. Without antibiotic treatment, about 56% of cells exhibited membrane-associated foci (Figure 10, black), while 34% displayed a mixture of diffuse signal and spots (Figure 10, dark gray). Approximately 10% of cells showed a completely diffusive signal (Figure 10, medium gray), and none of the cells (0%) displayed a signal concentrated on the chromosome (Figure 10, light gray). In contrast, cells treated with vancomycin did not display membrane-associated foci (Figure 10, black), while approximately 21% exhibited a diffuse signal with only a few spots (Figure 10, dark gray). 29% of cells showed a fully diffusive signal (Figure 10, medium gray), and 50% displayed a signal clearly concentrated on the nucleoids (Figure 10, light gray). These results reveal that treatment with vancomycin significantly alters the localization pattern of BacR, resulting in a large relocalization from the membrane onto the chromosome.

**Figure 10:**
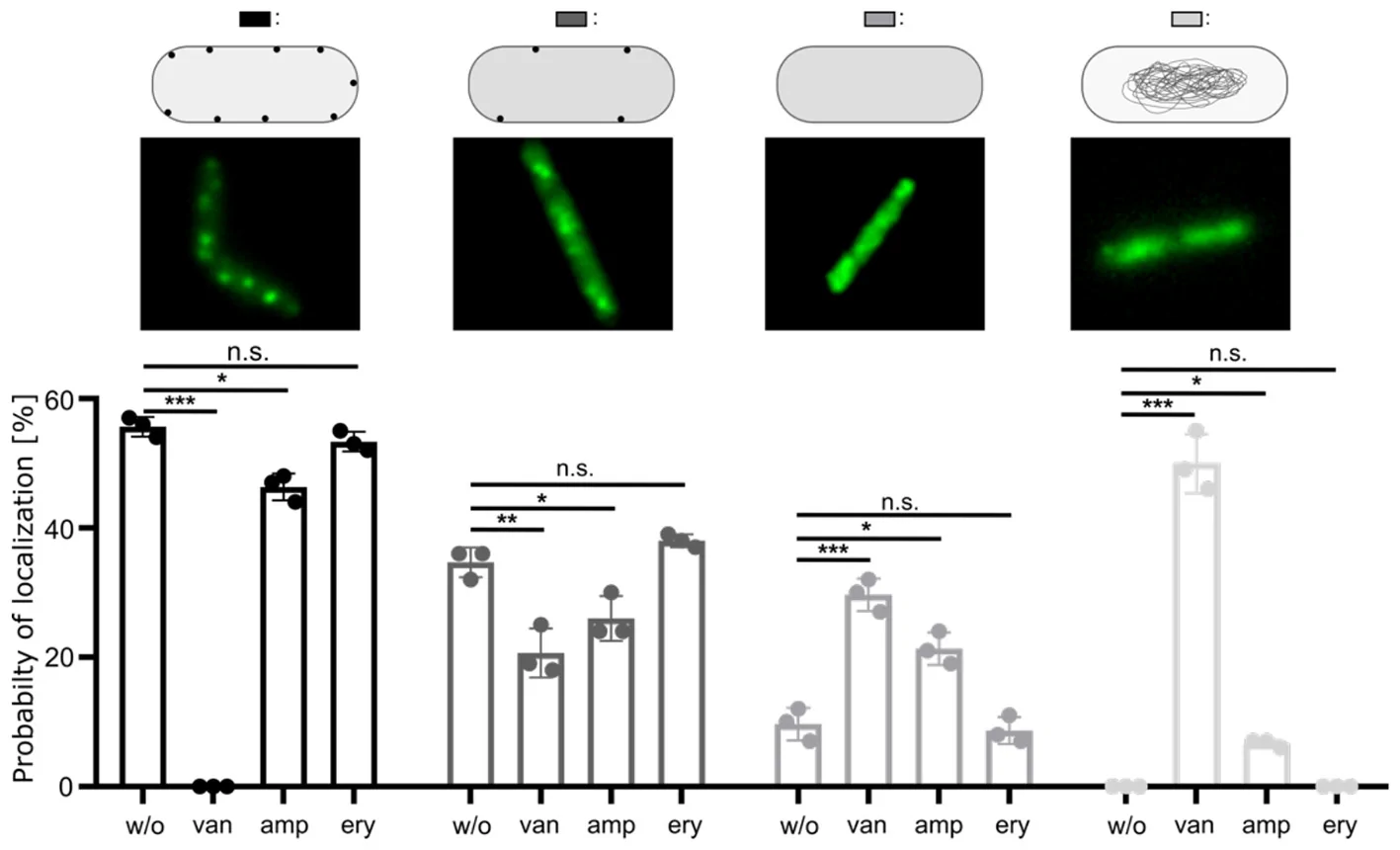
Quantification of localization pattern of antibiotic-treated 3610 DK1042 BacR-mNG cells. Upper panels: Epifluorescence microscopy of BacR-mNG, with cartoons illustrating the pattern of localization of BacR. Cells were incubated for 30 min without (w/o) treatment, with vancomycin (van), ampicillin (amp), or erythromycin (ery). The chromosome was stained with DAPI. Merge shows the overlay of BacR-mNG and DAPI. Scale bars 2 μm. Lower panel: bar plot shows the probability of the localization patterns of the cells in the epifluorescence images. Shown are examples of the most common localization pattern in every treatment. 394 cells w/o, 387 cells with van, 401 cells with amp, and 365 cells with ery treatment of the triplicate were analyzed. Error bars represent the standard deviation. Significance symbols *, **, and ***, represent *P*-values lower than 0.05, 0.01, or 0.001. Statistically not significant (n.s.; *p*-value > 0.05).

As a control, cells were treated with erythromycin, a macrolide antibiotic that targets the translational machinery by binding near the exit tunnel of the nascent peptide, thereby physically blocking the release of some newly synthesized polypeptides. Cells largely retained membrane-associated foci (Supplementary Figure S11), and no statistically significant changes in localization (Figure 10). Thus, BacR did not respond to general cellular stress, as opposed to vancomycin treatment. To test if BacR localization if affected by stress to the cell wall, we also treated cells with a sublethal concentration to ampicillin (Supplementary Figure S11). Quantification of the BacR localization pattern reveal a statistically significant change following addition of ampicillin (Figure 10). Relocalization of BacR-mNG from the membrane onto the nucleoids was significant, but not as pronounced compared with vancomycin-treated cells. These data suggest a rather specific response to cell wall stress.

### 2.8 Loss of BacR does not affect fitness related to sublethal doses of vancomycin

Because vancomycin treatment resulted in the most pronounced changes in BacR localization, we investigated whether BacR plays a role in cell survival under vancomycin-induced stress. For this, the colony-forming units (CFUs) of an in-frame *yhbD* deletion strain were compared to those of the wild type. Notably, the deletion strain showed increased survival during exponential growth (Figure 11A, dark gray), for which we have no explanation. The effect can not be explained by cell-chaining, for which there was no visible difference between wild type and mutant cells (Figure 11C). Also, different plating efficiency was not based on a higher growth rate, which was not significantly dissimilar between the strains (Figure 11B). Under vancomycin treatment, the deletion strain did not exhibit a statistically significant difference in vancomycin sensitivity (Figure 11A). These analyses suggest that *yhbD* does not influence survival under vancomycin-induced cell wall stress.

**Figure 11:**
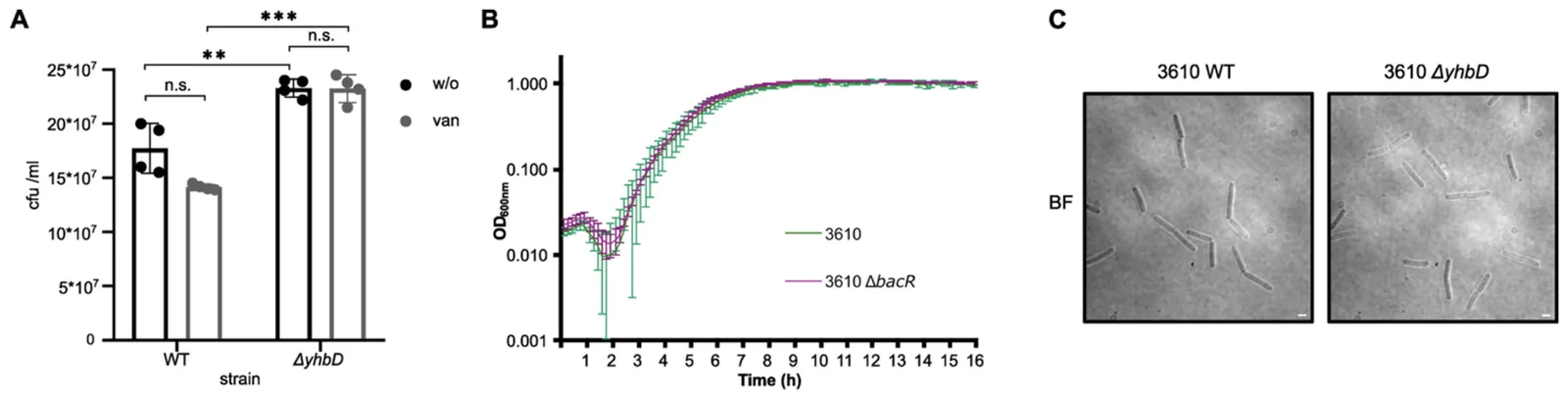
Survival and growth assays. (**A**) Survival assay of 3610 DK1042 wild type cells (WT) and of Δ*yhbD* mutant cells following vancomycin treatment. Bar plot shows the colony forming units (CFU) per ml of cells that were treated with or without (w/o) vancomycin (van) for 60 min, data represent four replicates. Error bars = SD. Significance symbols *, **, and *** represent *P*-values lower than 0.05, 0.01, and 0.001. Statistically not significant (n.s.; *p*-value > 0.05). **(B)** Grow curve showing optical density at 600 nm of wild type or mutant strains in LB medium. **(C)** Bright field (BF) images of cells from exponential phase. Scale bar 2 μm.

## 3 Discussion

The function of proteins containing the DUF4004 domain is unknown, except for a role of transcriptional regulator Cap0037 in metabolic adaptation in Clostridia. In our work, we characterize a second member of the class, BacR (YhbD), from *B. subtilis*, homologs of which are found in Bacilliota and Clostridia, and few archaea. We show that BacR plays a rather specific role in affecting transcript levels of a single operon, the *yhbDEF* operon, rather than a more global role as shown for Cap0037. Acting as a regulator of the expression of bactofilins, BacE and BacF, we propose to rename “YhbD” as BacR. By positively affecting mRNA levels of the *yhbDEF* operon, BacR also affects its own transcript levels; whether this occurs by affecting the activity of RNA polymerase or by e.g., mRNA stability, remains unclear at present. In case BacR acts as a genuine transcription factor, this would be similar to Cap0037, however in the opposing manner, because Cap0037 operates as a repressor of transcription. This highlights an apparent versatility of DUF4004 containing proteins with respect to their effect on transcription. However, in contrast to Cap0037, for which a specific DNA-binding motif is known (Nguyen et al., 2016), and with its identified binding sites overlapping with **–**35 or **–**10 regions in promoters, we have not been able to demonstrate specific DNA binding for BacR, but binding to a large variety of DNA sequences with no apparent preference, wherefore its effect on transcription may be direct or indirect. An additional, noteworthy difference between the two DUF4004-containing proteins is the genetic position of their genes, with the Ca*cap0037* gene being positioned on a plasmid, and *B. subtilis yhbD* on the genome. However, like *yhbD*, the *C. acetobotylicum* gene has kept the genetic link to a bactofilin gene, which can be found in many Bacilli and Clostridia. Thus, members of DUF4004 proteins are implicated in quite strikingly different aspects of transcriptional regulation, from chromosomal or plasmid contexts, and thus show a pronounced evolutionary plasticity.

Most importantly, our work unravels an intricate relation between BacR and bactofilins, in that the genetic connection to bactofilins is extended to physical protein/protein interactions. We found that BacR binds to both BacE and BacF, and that binding to bactofilins strongly interferes with DNA binding by BacR. Thus, there appears to be a partner-switching mechanism operating in *Bacillus* (extending the finding from *Geobacillus* proteins), where BacR either binds to bactofilins, or to the chromosome. This is translated into a somewhat spatially regulated process, because in the presence of bactofilins, BacR localizes to foci at the cell membrane, while the depletion of BacE and BacF leads to nucleoid-associated localization of the regulator. While the extent of membrane association remains uncertain, the bactofilin-dependent relocalization of BacR strongly supports a recruitment mechanism, reducing BacR's ability to interact with the chromosome (Figure 12). On the other hand, the depletion of bactofilins leads to a reduction of BacR within the cell (Figure 9), indicating that bactofilins may stabilize BacR, possibly by sequestering it from cellular proteases. Thus, our work establishes a direct link between a DUF4004 ortholog and bactofilins, which may extend to many if not most other members of the protein family, as some are also found encoded upstream of a bactofilin gene.

**Figure 12:**
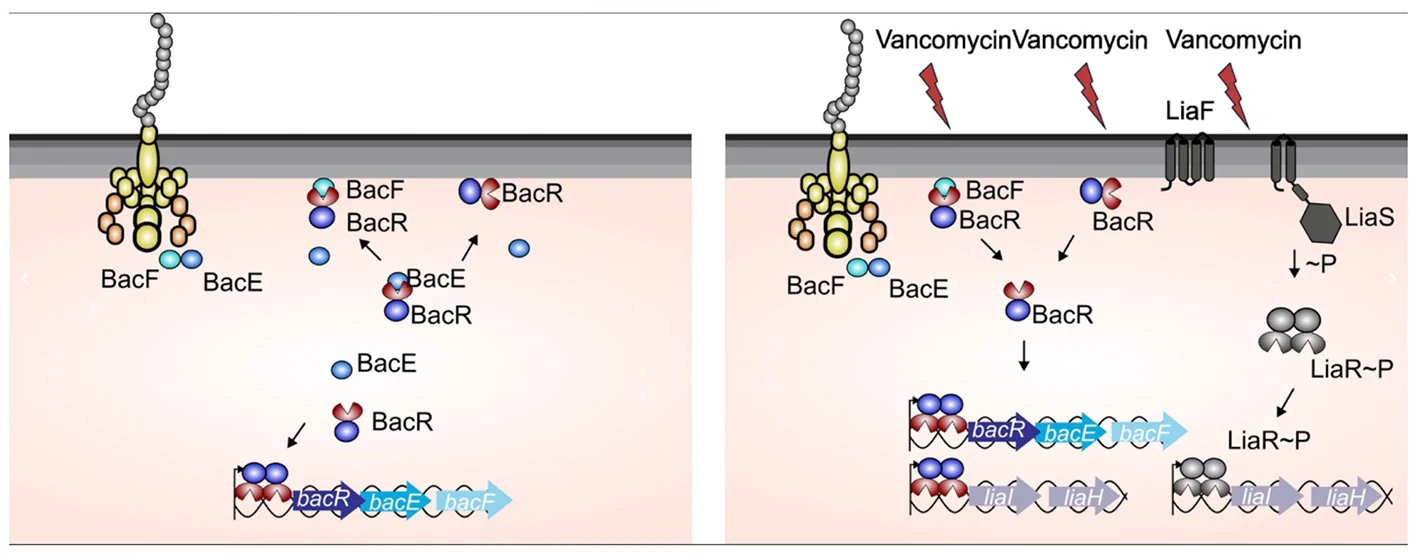
**Model** for the putative partner switch mechanism of transcriptional regulation of the *yhb*/bactofilin operon by BacR, and its proposed role in sensing of cell wall stress.

What could be the purpose of the bactofilin-mediated (membrane) association of BacR and its interaction with BacE and BacF? We have not been able to label both, BacR and bactofilins, to prove that they co-localize (possibly because tagging of two partners in the complex interferes with proper localization), but given that the localization pattern of BacR in exponentially growing cells is strikingly similar to that of bactofilins, and their direct interaction *in vitro*, it seems highly likely that BacE and BacF are directly responsible for membrane recruitment of BacR. This leads to the model that bactofilins recruit BacR to the cell membrane and inhibit its binding to DNA, downregulating transcription of *yhbDEF* (Figure 12). On the other hand, BacR was also found to be present in the cytosolic fraction of cells, even in the presence of bactofilins, showing that to some degree, BacR is able to bind to the chromosome and thus to the *yhbDEF* region. In parallel, bactofilins stabilize BacR. Cycling between membrane-associated bactofilins and the chromosome likely fine-tunes transcription levels of bactofilins, and leads to an overall low transcript level of the operon.

Like many transcription factors, BacR was found as a dimer in solution, and showed binding to double stranded DNA. Quite unusually, BacR featured much higher affinity to large DNA fragments than to shorter ones, hampering our attempts to narrow down possible specific binding sites within the *yhbDEF* operon. With regard to a possible function, we have found that membrane-association of BacR is completely lost when cells are treated with sublethal concentrations of the antibiotic vancomycin, targeting cell wall crosslinking. While we can not rule out that the oberved membrane-localization pattern of BacR is exaggerated in our cell biological analyses, BacR is entirely nucleoid-associated following vancomycin treatment, and to a lesser degree also following ampicillin treatment, which inhibits cell wall crosslinking Penicillin-binding proteins. Examples for transcription-associated factors being reseased from the membrane to interact with promoters are sigma factors, e.g., during sporulation in firmicutes (Al-Hinai et al., 2015) and ECF anti-sigma factors, which respond to e.g., cell wall stress (Hughes and Mathee, 1998). To the best of our knowledge, there is no report of a genuine transcription factor that is released from the cell membrane upon a specific stimulus, as we propose for BacR. No such relocalization for BacR was observed upon induction of general stress, indicating a rather specific response to cell wall stress. Based on a similar relocation effect caused by the depletion of bactofilins, it is attractive to think that the vancomycin-induced effect on BacR localization could be mediated by the interaction with membrane-associated bactofilins. However, because BacR becomes generally much less abundant in the absence of bactofilins, this idea is difficult to prove experimentally.

Thus, potentially, BacR could mediate the signaling transfer of stress for the cell wall synthesis machinery to an intracellular response, in parallel to the Lia system. In this three component system, LiaF restrains the membrane integral histidine kinase LiaS (Figure 12), preventing unwanted activation of the stress response. Cell wall stress induced by Bacitracin or Vancomycin relieves the inhibition by LiaF, such that LiaS auto-phosphorylates and passes the phosphate group to LiaR (Jordan et al., 2006). Phosphorylated LiaR binds to target promoters, inducing e.g., the synthesis of the LiaH phage shock protein that helps stabilize the membrane (Dominguez-Escobar et al., 2014). It will be interesting to investigate if BacR and bactofilins constitute an additional line of defense against cell wall stress that could be redundant with the Lia system.

In toto, our work extensively deepens the knowledge on proteins containing the DUF4004, and reveals that this class of proteins displays illustrious properties, comprising elements from subcellular relocalization events to partner switch events including DNA and proteins, specifically bactofilins, which are highly conserved in a wide range of bacteria. Our analyses show that further investigation of DUF4004 proteins including BacR will be rewarding in terms of discovery of molecular mechanisms, and awaits more research showing the importance of BacR for the physiology of *B. subtilis* cells.

## 4 Material and methods

### 4.1 Bacterial growth conditions

*E. coli* and *B. subtilis* strains were cultivated in lysogeny broth (LB) medium or on 1.5% LB agar plates, according to the manufacturer's instructions (Carl Roth GmbH, Germany). Liquid cultures were grown at 200 rpm, at 37 °C for *E. coli* and 30 °C for *B. subtilis*, unless stated otherwise. For plasmid propagation and molecular cloning, *E. coli* strains were grown at 37 °C in LB medium supplemented with 100 μg/mL ampicillin or 50 μg/mL kanamycin, depending on the plasmid. For heterologous protein expression, overnight cultures were inoculated from fresh LB agar plates and grown in LB medium containing 1% lactose at 30 °C. Transformation with expression plasmids was performed one day prior to induction. *B. subtilis* strains were cultivated at 30 °C in liquid LB medium or on LB agar plates containing 5 μg/mL chloramphenicol, or 25 μg/mL kanamycin, or 100 μg/mL spectinomycin. To verify the successful integration at the *amyE*-locus in *B*. *subtilis*, LB-Agar plates were supplemented with 1% starch (w/v), and analyzed for loss of halo formation. For the analysis of biofilm formation, defined MSgg agar plates were used. MSgg medium (per 100 mL) was composed of: 1 ml 0.5 M potassium phosphate buffer (prepared from 6.12 g K_2_HPO_4_ x 3 H_2_O and 3.16 g KH_2_PO_4_ in 100 mL, pH 7.0), 10 ml of 1 M MOPS (pH 7.0), 1 mL 50% glycerol, 1 mL 50% L-glutamate monopotassium monohydrate, 0.1 mL 0.7 M CaCl_2_ x 2 H_2_O, 0.1 mL 2 M MgCl_2_ x 6 H_2_O, 0.1 mL 0.1 M FeCl_2_ x 6H_2_O, 0.1 mL 0.05 M MnCl_2_ x 4H_2_O, 0.001 mL 0.1 M ZnCl_2_, 0.002 mL 0.1 M thiamine-HCl, and 1.5 g Bacto agar. For motility assays, LB plates containing 0.3% Bacto agar were used to allow swimming motility of *B. subtilis* cells. To investigate the cellular response to antibiotic stress via fluorescence microscopy or survival assay, cultures were grown to an optical density at 600 nm (OD_600_) of 0.5. Cells were then incubated for 30 min with one of the following antibiotics at the indicated final concentrations: 4 μg/mL vancomycin, 1.2 μg/mL ampicillin, or 1.5 μg/mL erythromycin. Subsequently, the cells were either directly subjected to microscopy or spotted onto 1.5% LB agar plates for the survival assays.

### 4.2 Strain construction

Oligonucleotides and generated plasmids are listed in Supplementary Tables S1 and S2, respectively. PCR fragments were generated by using chromosomal DNA of *B. subtilis* 3610 DK1042 (wild type), *G. thermodenitrificans* wild type cells, *B. subtilis* 3610 BacR-mNG, or vectors pSG1164-mNG and pSG1193 (Supplementary Table S2). For tagging of *yhbD* at its native chromosomal locus, the integrative plasmid pSG1164 (Feucht and Lewis, 2001) was used, modified to carry an mNeonGreen (mNG) fluorescent reporter instead of the original YFP tag. The final 500 base pairs of the *yhbD* coding sequence, excluding the native stop codon, were amplified by PCR using primers containing restriction sites for *Eco*RI and *Bam*HI (Supplementary Table S1). The PCR product was ligated into pSG1164 in-frame with the mNG coding sequence via a flexible linker which encodes for 14 amino acids. The resulting construct allowed for integration of the fusion at the native *yhbD* locus under control of the endogenous promoter. The downstream genes *bacE (yhbE)* and *bacF (yhbF)*, located in the same operon, were placed under control of a xylose-inducible promoter, which is activated in the presence of xylose and repressed by glucose.

To integrate the complete *yhbD* gene, including its native promoter and a C-terminal mNeonGreen (mNG) tag, into the *amyE* locus, genomic DNA from strain 3,610 BacR-mNG was used as a template. A DNA fragment comprising approximately 250 base pairs upstream of *yhbD* (including the putative native promoter), the entire *yhbD* coding sequence, the bases encoding the 14-amino-acid linker, and the mNG coding region was amplified by PCR. The insert was cloned into the integrative vector pSG1193 (Feucht and Lewis, 2001) using Gibson Assembly (New England Biolabs GmbH, USA). To facilitate this, the pSG1193 (Feucht and Lewis, 2001) vector backbone was PCR-amplified in a way that removed the xylose-inducible promoter originally present in the vector (Supplementary Table S1), thereby allowing expression of *yhbD*-mNG under control of the native *yhbD* promoter. The resulting construct enabled stable chromosomal integration of the fusion at the *amyE* locus.

To generate genomic DNA (gDNA) for the *yhbD::kan* allele, a chromosomal transformation was performed using gDNA from the *B. subtilis* BKK01660 strain (Koo et al., 2017). For construction of a markerless in-frame deletion of *yhbD*, the protocol provided by the *Bacillus* Genetic Stock Center (BGSC, Wisconsin, USA) was followed. This method is based on the use of the loop-out plasmid pDR244 to excise a loxP-flanked antibiotic resistance cassette. First, pDR244 was transformed into the BKK01660 strain carrying the loxP-flanked kanamycin resistance cassette, and transformants were selected on LB agar supplemented with spectinomycin. Several spectinomycin-resistant colonies were then transferred to non-selective LB plates and incubated overnight at 42 °C to induce plasmid loss and promote Cre-mediated recombination. Following incubation, colonies were screened for spectinomycin sensitivity to confirm loss of the pDR244 plasmid, and for erythromycin sensitivity to verify excision of the resistance cassette. Colonies were restreaked to obtain single clones, and successful deletion of the cassette was confirmed by diagnostic PCR. Δ*yhbD::kan* and Δ*yhbD* deletion strains were generated by SSP1 transduction. Transfer of chromosomal DNA of *B. subtilis* BKK01660 strain was performed by SPP1-mediated phage transduction to the recipient strains. Phage lysates were prepared by mixing SPP1 phage stocks (in dilutions) with a bacterial culture. Infected culture was incubated for 15 min at 37 °C. Sample was supplemented with TY soft agar (0.3%) and plated on a 1.5% TY-plate. Plaque formation can be detected after 16 h of incubation at 30 °C. TY-medium was added to the plate and phages were harvested by collecting the soft agar/ liquid medium mixture. Samples were centrifuged and supernatant was filtered through a 0.2 μm pore size syringe filter. For transfection, 1 mL of the respective stationary grown *B*. *subtilis* recipient strain was inoculated with 30 μL of the phage lysate in 10 mL TY media (5 g/L NaCl, 5 g/L yeast extract, 10 g/L tryptone, 1 mM MgSO_4_, 10 μM MnSO4). The mixture was incubated on a shaking platform for 30 min at room temperature, followed by a centrifugation step to sediment the cells. The pellet was resuspended in approximately 100 μL of the remaining supernatant and plated on antibiotic containing LB agar plates supplemented with 10 mM sodium citrate to prevent phage re-infection. To generate the Δ*yhbD* was used the protocol provided by the BGSC as described above.

### 4.3 Generation of recombinant plasmids

To construct a plasmid for heterologous overexpression of GST-tagged BacR in *E. coli*, the complete *yhbD* coding sequence from *B. subtilis* was amplified by PCR using primers containing *Nco*I and *Xho*I restriction sites (Supplementary Table S1), and was cloned into expression vector pGAT3. The resulting recombinant plasmid allowed for expression of an N-terminal GST fusion of BacR in *E. coli*. To generate a plasmid for heterologous overexpression of an N-terminally StrepII-tagged BacR protein from *G. thermodenitrificans*, the complete *yhbD* coding sequence was amplified by PCR using primers introducing *Nhe*I and *Xho*I restriction sites (Supplementary Table S1) and was cloned into expression vector pET24d-StrepII, which encodes a StrepII tag followed by a twelve-amino-acid flexible linker upstream of the cloning site.

### 4.4 Protein purification

For protein purification plasmids were transformed the day prior the experiment to C41 cells and six liters of LB-media were incubated with cells over night at 30 °C on a shaking platform (120 rpm) with 1% lactose for protein overexpression (for all used constructs). Cells were harvested at 4,000 rpm for 15 min and the generated cell pellet was resuspended in 10 ml of lysis buffer per gram of cells. Depending on the construct, different buffer compositions were used: GST-*Bs*BacR (50 mM Tris-HCl, 600 mM KCl, 250 mM NaCl, 5 mM DTT, 10% glycerol, 0.5 mg/mL DNAse, 0.5 mg/mL RNAse); His6-GB1-*Gt*BacE and His6-GB1-*Gt*BacF (20 mM Hepes, 250 mM NaCl, 40 mM imidazole, 20 mM KCl and 20 mM MgCl_2_, 0.5 mg/mL DNase, 0.5 mg/mL RNase), and StrepII-*Gt*BacR (20 mM Hepes, 250 mM NaCl, 20 mM KCl and 20 mM MgCl2, 0.5 mg/mL DNase, 0.5 mg/mL RNase). To disrupt the cells, a microfluidizer (microfluidics) was used and cell debris was removed by centrifugation at 15,000 rpm for 20 min. Filtered lysate (0.45 μm, Filtropore) was subsequently applied to 3 sequentially mounted GSTrap™ HP colums (GE Healthcare), 5 mL HIS-trap column (GE Healthcare) or 5 mL StrepTrap HP column (GE Healthcare) which was further pre-equilibrated with the respective buffer. After applying the sample to the column, a washing step followed before the elution with elution buffer for GST-*Bs*BacR (50 mM Tris-HCl, 600 mM KCl, 250 mM NaCl, 5 mM DTT, 10% glycerol, 20 mM reduced glutathione), His6-GB1-*Gt*BacE and His6-GB1-*Gt*BacF (20 mM Hepes, 250 mM NaCl, 250 mM imidazole, 20 mM KCl and 20 mM MgCl2) and for StrepII-*Gt*BacR (20 mM Hepes, 250 mM NaCl, 20 mM KCl and 20 mM MgCl2, 2.5 mM desthiobiotin). To concentrate the protein a Amicon Ultra-10K 0.5 mL or Amicon Ultra-30K 0.5 mL (Millipore) was used, depending on the size of the protein. Protein concentration was estimated with a NanoDrop™ Lite spectrophotometer (Thermo Scientific). Fractions were analyzed by sodium dodecyl sulfate–polyacrylamide gel electrophoresis (SDS–PAGE).

### 4.5 Analytical size exclusion chromatography

Size exclusion chromatography was used to analyze oligomeric state of proteins from concentrated elution fraction. For this we used a NGC-system (Bio-Rad) with Superdex 200 Increase 10/300 column (Cytivia). Before use, the column was equilibrated with the following buffer: 20 mM Hepes, 250 mM NaCl, 20 mM KCl and 20 mM MgCl2 (pH 8.0). For calibration a standard protein mix was used with known molecular weights (Thyroglobulin (bovine) 670 kDa; g-globulin (bovine) 15 kDa 8; Ovalbumin (chicken) 44; Myoglobin (horse) 17; Vitamin B12 1.35 kDa; Biorad). Chromatograms were generated with the NGC software. Fractions were analyzed by SDS-PAGE.

### 4.6 Mass photometrie

Mass photometry (MP) analysis was performed using a TwoMP mass photometer (Refeyn Ltd, UK). The instrument was operated with the AcquireMP v2023 R1.1 software (Refeyn Ltd, UK). Microscope coverslips (22 mm × 50 mm, No. 1.5, Marienfeld) and CultureWell Reusable Gaskets (CW-50R-1.0, 3 mm × 1 mm; Grace Bio-labs, USA) were cleaned three times with isopropanol and ultrapure-grade water (Milli-Q, Merck, Germany), air-dried with a compressed air stream, assembled, and mounted with immersion oil (Immersol™ 518F; Carl Zeiss, Germany). For one MP measurement, a drop of 18 μL of HEPES buffer (20 mM HEPES, 250 mM NaCl, 20 mM KCl and 20 mM MgCl2, pH 8.0) was applied to one well of the gasket and focused via the “Droplet dilution” option. Prior to each measurement, protein solutions were freshly pre-diluted to a concentration of 100 nM with HEPES buffer. For the MP measurements, 1.0 μL of pre-diluted protein mixture was applied to the drop. The MP instrument was calibrated with a custom molecular weight standard (84–336 kDa) that was pre-diluted with HEPES buffer. MP measurements were recorded for 60 s at 100 frames per s and analyzed with the DiscoverMP v2023 R.1.2 software (Refeyn Ltd, UK). Peaks were fitted with a Gaussian fit, and the total number of counts was predicted by the Gaussian model.

### 4.7 Electromobility shift assays (EMSA)

To determine whether the recombinant proteins can bind single- or double-stranded DNA, an electromobility shift assay (EMSA) was performed. The promoter region of the *yhbDEF* operon were predicted to lie within the *cspR* gene by BPOM tool (http://www.softberry.com). For the EMSA, 20 μL of reaction mixture containing 200 ng of the respective DNA, EMSA buffer (10 mM Tris, 40 mM NaCl, 5 mM KCl, 5% glycerol, 2 mM MgCl2, and 0.025 mg/l BSA, pH 8.0), recombinant protein (2–16 μM), and elution buffer was mixed and incubated at 20 °C for 20 min. Subsequently, 4 μL of Purple No-SDS-Gel-Loading-Dye (NEB) was added to each sample, and the mixture was loaded onto a native 6% acrylamide gel. For detection, gels were stained with Midoori-Green for approximately 20 min or imaged using the ChemiDoc Touch Imaging System. Primers used for generating the DNA fragments are listed in Supplementary Table S1. For the generation of 42 bp 6-Carboxyfluorescein (6-FAM)-labeled dsDNA, primer annealing was performed for 3 min (95 °C, followed by 0 °C). To investigate DNA binding in the presence of His-GB1-*Gt*BacE (16 μM) or His-GB1-*Gt*BacF (16 μM), StrepII-*Gt*BacR was incubated for 20 min at 22 °C with slow rotation before DNA incubation.

### 4.8 Pulldown assays

StrepTagII pulldown assays were conducted to study protein interactions. Therefore, StrepII-tagged protein cultures and tested His-tagged protein cultures (400 ml) were combined and lysed following the procedures outlined above. Subsequently, the lysates were incubated with 30 μL of MagStrep Strep-Tactin XT beads (Iba Life Sciences) for 30 min at 22 °C with gentle rotation. Following centrifugation (4,000 rpm, 5 min, 22 °C), the supernatant was discarded, and the beads underwent three washes with 500 μL of SEC Buffer, utilizing a magnetic rack. The proteins bound to the beads were eluted using 200 μM D-Biotin in HEPES buffer (20 mM Hepes, 250 mM NaCl, 20 mM KCl and 20 mM MgCl_2_; pH 8.0) and then subjected to analysis through western blots.

### 4.9 Cell fractionation

Strains were cultivated in LB medium (30 °C) with the addition of the specific antibiotic until exponential growth phase. 50 mL of the cells were harvested and resuspended in appropriate volume of lysis buffer (50 mM EDTA, 100 mM NaCl, pH 7.5). Buffer was supplemented with Protease Inhibitor Cocktail complete™: EDTA-free Protease Inhibitor Cocktail (Roche), and lysozyme (2.5 mg/mL). Lysates were generated by centrifugation. For protein fractionation of the cell lysate, first, a 50 μL sample was withdrawn for SDS-PAGE analysis. The remaining cell lysate was transferred to a centrifugation tube with a volume capacity of 4 mL (Beckman Coulter) Samples were centrifuged in 4 mL, Quick-Seal, g-Max Polypropylene tubes (Beckman Coulter) and were filled up with lysis buffer F to ¾ of the total tube volume (dilution about three-fold). We used an ultracentrifuge (Optima XPN 80, Beckman Coulter), and a 70 Ti fixed-angle rotor. After centrifugation at 100,000 g for 1 h in the ultra-centrifuge a 50 μL sample of the cytosol containing supernatant was taken. The remaining supernatant was discarded. The remaining pellet was resuspended in 1 ml lysis buffer. Again a 50 μL sample (which is about three-fold concentrated relative to the cytosolic fraction) was taken for SDS-PAGE analysis. Resulting fractions were termed “lysate,” and after ultra-centrifugation, the supernatant (cytosolic proteins) “cytosol,” and the pellet containing membrane proteins “membrane”. As a control, wild type strain PY79 and 3,610 was used. For the quantification of the intensity of the proteins on the western blot the Biorad tool was used.

### 4.10 Survial assays

Freshly restreaked *Bacillus subtilis* strains were used to inoculate an LB overnight culture supplemented with the appropriate antibiotics. On the following day, cultures were diluted to an OD600 of 0.1 in fresh LB medium and incubated at 37 °C with shaking at 200 rpm until an OD600 of approximately 0.5 was reached. The cultures were then split into two aliquots: one was left untreated, while the other was supplemented with vancomycin at a final concentration of 4 μg/mL. Both aliquots were incubated for an additional 1 h under the same conditions. After incubation, cells were serially diluted (1:10) up to a final dilution of 10^−6^. A volume of 100 μL from the 10^−3^ to the 10^−6^ dilutions was plated onto LB agar plates. Colony-forming units (CFU) were determined by counting visible colonies and calculating the average CFU per milliliter. This was done by multiplying the number of colonies by the corresponding dilution factor and dividing by the plated volume (0.1 mL). Statistical analysis of survival rates was performed both within and between genotypes (wild type and mutant). Arithmetic means and standard deviations from three biological replicates were used for all comparisons. To compare wild type and mutant under identical treatment conditions (Untreated or Vancomycin-treated), the Welch's *t*-test for unpaired samples was applied, as this method is robust against unequal variances and particularly suitable for biological datasets with small sample sizes. For multiple comparisons, the potential inflation of the type I error rate was considered, and where applicable, the significance threshold was adjusted using methods such as Bonferroni correction. Interpretation of statistical results was based on *p*-values as well as the magnitude of mean differences and, where relevant, calculated effect sizes to assess biological relevance. Graphical representation of the data, including bar charts with error bars, was carried out using GraphPad Prism (Version 8.0.1, GraphPad Software, San Diego, CA, USA).

### 4.11 RNA-Sequencing for transcriptome analysis

Samples for transcriptome analysis were obtained in triplicates from *Bacillus subtilis* 3610 wild type (wt) and the deletion mutant 3610Δ*yhb*Dm, either untreated or treated with vancomycin. Precultures of wt and mutant strains were started from a single colony in LB medium, incubated in a water bath at 37 °C with shaking at 220 rpm until reaching an OD_600_ of 1. Cultures were then diluted to an OD_600_ of 0.1 in fresh prewarmed LB medium (main cultures). At an OD_600_ of 0.5, each main culture was split into two pre-warmed flasks: one served as the untreated control, while the other was supplemented with vancomycin (4 μg/mL final concentration). Both cultures were incubated for additional 30 min. For harvesting, 4.5 mL of culture were taken from each flask and immediately mixed with 500 μL of ice-cold stop solution (95% ethanol, 5% phenol, pH 5.5). Samples were snap-frozen in liquid nitrogen to arrest cellular processes and stored at −80 °C until further processing. Replicates were obtained on three different days, each time starting with one pre-culture of the wild type and the mutant strain.

Samples were thawed on ice and pelleted by centrifugation for 10 min at 4 °C at 4,000 rcf. For total RNA isolation, the Qiagen miRNeasy Kit was used. Cell pellets were resuspended in 1 mL of QIAzol lysis reagent and homogenized with the precellys 24 tissue homogenizer using Lysing Matrix B containing 0.1 mm silica beads (MP biomedicals) and the following settings: 2 x 30 s 5,800 rpm, 20 s break. The supernatant was transferred into a new tube and RNA isolation was performed according to the manufacturer's instruction including the optional on column DNaseI digestion. RNA integrity was assessed using fragment analyzer Agilent DNF-471 RNA Kit (15 nt). Library preparation and sequencing was done by GENEWIZ by Azenta Life Sciences. RNA samples were quantified using Qubit 4.0 Fluorometer (Life Technologies, Carlsbad, CA, USA) and RNA integrity was checked with RNA Kit on Agilent 5,600 Fragment Analyzer (Agilent Technologies, Palo Alto, CA, USA). RNA sequencing and library preparation was prepared using NEBNext rRNA Depletion Kit (Bacteria) and NEBNext Ultra II Directional RNA Library Prep Kit for Illumina following manufacturer's instructions (NEB, Ipswich, MA, USA). Ribosomal RNA was depleted with NEBNext rRNA Depletion Kit (Bacteria). rRNA depleted RNAs were fragmented. First strand and second strand cDNA were subsequently synthesized. The second strand of cDNA was marked by incorporating dUTP during the synthesis. cDNA fragments were adenylated at 3′ends, and indexed adapter was ligated to cDNA fragments. Limited cycle PCR was used for library amplification. The dUTP incorporated into the cDNA of the second strand enabled its specific degradation to maintain strand specificity. Sequencing libraries were validated using DNA Kit on the Agilent 5,600 Fragment Analyzer (Agilent Technologies, Palo Alto, CA, USA), and quantified by using Qubit 4.0 Fluorometer (Invitrogen, Carlsbad, CA). The sequencing libraries were multiplexed and clustered on the flowcell. After clustering, the flowcell was loaded on the Illumina NovaSeqX Plus instrument according to manufacturer's instructions. The samples were sequenced using a 2 x 150 Pair-End (PE) configuration. Image analysis and base calling were conducted by the NovaSeq Control Software v1.8 on the NovaSeqX Plus instrument. Raw sequence data (bcl files) generated from Illumina NovaSeqX Plus was converted into fastq files and de-multiplexed using Illumina bcl2fastq program version 2.20. One mismatch was allowed for index sequence identification.

Raw sequencing reads were assessed for quality using FastQC (v0.12.1, Babraham Bioinformatics, Babraham Institute, Cambridge, UK) prior to alignment. Adapter trimming was not performed as STAR performs soft-clipping during alignment. Reads were aligned to the *Bacillus subtilis* NCIB 3610 reference genome (GenBank accession CP020102.1) using STAR (v2.7.11a) (Dobin et al., 2013), executed within the Galaxy Europe platform (usegalaxy.eu) (Afgan et al., 2018). Read quantification was performed using featureCounts (v2.1.1) (Liao et al., 2014) with reverse strand specificity, paired-end mode requiring both reads for assignment, chimeric fragments excluded, primary alignments only, and a minimum mapping quality of 10.

Differential gene expression analysis was performed in R (v4.4.1) using DESeq2 (v1.44.0) (Love et al., 2014) with the design formula ‘~ cell_type ^*^ condition'. Wald tests were used to assess differential expression across five contrasts: (i) WT vancomycin vs. control, (ii) Δ*yhbD* vancomycin vs. control, (iii) Δ*yhbD* vs. WT under control conditions, (iv) Δ*yhbD* vs. WT under vancomycin treatment, and (v) the genotype × treatment interaction term. Log2 fold changes were shrunk using the adaptive shrinkage estimator (ashr; CRAN.R-project.org/package=ashr). Genes were considered differentially expressed at an adjusted p-value < 0.1 (Benjamini-Hochberg correction) and |shrunk log2FC| > 1. Data visualization was performed using ggplot2, ggrepel (CRAN.R-project.org/package=ggrepel), ComplexHeatmap (Gu, 2022), and patchwork (CRAN.R-project.org/package=patchwork).

Raw sequencing data and count matrices have been deposited in the NCBI Gene Expression Omnibus (GEO) under accession number GSE326318 (reviewer token: ipylgkeidlmjnwv). Analysis scripts are available at doi: 10.5281/zenodo.19309427. The Galaxy workflow used for alignment and quantification is available at https://usegalaxy.eu/u/patrikb/w/rnaseq-analysis-delta-yhbd-galaxy-workflow.

### 4.12 Epifluorescence microscopy

*B. subtilis* cells were grown at 30 °C and 200 rpm to the exponential phase and treated simultaneously with 0.5% glucose or 0.5% xylose (3610 YhbD-mNG). For wide-field epifluorescence microscopy, a Zeiss Observer A1 microscope (Carl Zeiss) equipped with an oil immersion objective (100 × magnification, 1.45 numerical aperture, Alpha PlanFLUAR; Carl Zeiss) was used. Images were captured with a charge-coupled device (CCD) camera (CoolSNAP EZ; Photometrics), and fluorescence excitation was provided by an HXP 120 metal halide lamp with intensity control. For sample preparation, round coverslips (25 mm, Marienfeld) were used, and 5 μL of cell culture was placed onto a 1.5% agarose pad. The agarose pads were prepared by sandwiching 100 μL of melted agarose between two smaller coverslips (12 mm). Images were processed using ImageJ. A YFP filter set (ET500/20 EX, T515lp, ET535/30 EM) was used for epifluorescence. For DAPI staining, 1 μL of a 10 mg/ml DAPI stock solution was added to 100 μL of cell culture shortly before microscopy. DAPI (4′,6-diamidino-2-phenylindole) specifically binds to A-T rich regions of double-stranded DNA and is commonly used to visualize the chromosome or nucleoid in bacteria. Upon UV excitation (358 nm), DAPI emits a blue fluorescence signal at 461 nm. For image acquisition, an iBeam smart 515 laser (F26–510HC-Basic DAPI) was used.

### 4.13 Single molecule tracking (SMT)

Microscopy was performed in LB medium with prior cultivation of *B. subtilis* at 30 °C and 200 rpm. Individual molecules were tracked using a custom-made slim-field setup on an inverted fluorescence microscope (Nikon Eclipse Ti-E, Nikon Instruments Inc.). An EMCCD camera (ImagEM X2 EM-CCD, Hamamatsu Photonics KK) was used to ensure high-resolution detection of the emission signal, resulting in a calculated resolution of the position of the molecule down to 20 nm. The central part of a 514 nm laser diode (max power 100 mW, TOPTICA Beam Smart) was used with up to 20% of the intensity (about 160 W cm^−2^ in the image plane) to excite samples fused to mNeonGreen, using a laser filter set (BrightLine 500/24, dichroic mirror 520, and BrightLine 542/27), by focusing the beam onto the back focal plane of the objective. A CFI Apochromat objective (TIRF 100 × Oil, NA 1.49) was used in the setup. Generated data was further analyzed by ImageJ (Bleaching curves). For the analysis, a video of 4,000 frames at 20 ms was recorded, with frames starting after 300 to 800 frames, depending on the time point when single molecule levels were reached due to bleaching, used for analysis (Hammerl and Graumann, 2024). The software Oufti (Paintdakhi et al., 2016) was used to set the necessary cell meshes, and Utrack (Jaqaman et al., 2008) was employed for automatic determination of molecule trajectories by setting a minimal track length of 5. The movement of tracks was then analyzed by the SMTracker software (Oviedo-Bocanegra et al., 2021) by using the SQD which describes the cumulative probability function of squared displacements. The localization of errors was calculated by using the mean square deviation analyses from the intercept of the graph with the y axis. For the sample preparation round coverslips (25 mm) were used and covering 5 μL cell culture with a 1.5% agarose pad. The agarose pads were made with water by sandwiching 100 μL of the melted agarose between two smaller coverslips (12 mm).

## Data Availability

The raw sequencing data and count matrices have been deposited in the NCBI Gene Expression Omnibus (GEO) under accession number GSE326318. The raw images for flouresecence microscopy are available upon request.
