# Supplementary material for: DUF4004 protein BacR (YhbD) of Bacillus subtilis is a regulator of bactofilin expression and interacts with bactofilins in a switch-like manner
Source: Front Microbiol. 2026 Sep 25;17:1922879. doi: 10.3389/fmicb.2026.1922879 (PMC13649987; doi:10.3389/fmicb.2026.1922879)
Supplement: Supplementary file 1 [file Supplementary_file_1.docx]

Supplementary Material

**A**

**B**

**Figure S1: Genetic neighborhood and phylogenetic tree of BacR (BacR) sequences. A)** Examples of genetic contexts for *yhbD/cap0037*. **B)** Phylogenetic tree of BacR sequence from *B. subtilis* and from other bacteria. Sequences were selected by UniProt pBLAST (Uniprot, 2019) on NCBI data base and the phylogenetic tree was generated by iTOLv7 (1). BacR protein from *B. subtilis* is shown in red.

**Fig. S2 Confidence metrics as the predicted local distance difference test (pLDDT) scores, which indicate the model's confidence in the predicted structure.** A-C) Fig. 1B-C: A) BsBacR monomer, B) GtBacR monomer, C) CaCap0037 monomer, D-E) Fig. S6: D) BsBacR dimer, E) GtBacR dimer, F-G) Fig. 5A-B: F) StrepGtBacR + His6-GB1GtBacE,  G) StrepGtBacR + His6-GB1GtBacF. H) The N-terminal 25 residues of *B. subtilis* YhbD (P97032) were excluded as a probable start-codon mis-annotation. The remaining sequence and *G. thermodenitrificans* YhbD (A0ABY9Q8P1) were predicted with ESMFold v1 and superposed with TM-align at the RCSB alignment server, giving a backbone RMSD of 1.70 Å over 208 aligned residues at 46.6% sequence identity.

**Figure S3: Quality analyses of transcriptomics. A)** heat maps of differences between replicates, **B-D)** grouping of data sets as exemplified by the *yhbDEF* operon.

**Figure S4: Volcano plots of changes seen in global transcription levels,** between exponentially growing cells and cells stressed by Vancomycin for 30 minutes. **A)** wild type cells, **B)** *yhbD* mutant cells.

**Figure S5: Purification and characterization of GST-*Gt*BacR.** Coomassie-stained SDS Page of purification steps of Strep-*Gt*BacR (~ 29 kDa) by streptavidin chromatography. Lane 1) represents Pre-stained PageRuler, 2) cells, 3) lysed cells, 4) lysate, 5) flow through, 6) wash and 7-10) elution fractions.

**Figure S6: AlphaFold prediction of dimers of BacR proteins**. **A-B)** Prediction of BsBacR Dimer (dark blue and light blue) (A) and GtBacR Dimer (dark green and light green) (B). Prediction is carried out by AlphaFold 3.0 and visualized by PyMOL 3.1.3. The black box labels the possible dimerization region, and the helix-turn-helix motif is highlighted in red. C Alignment of the Dimers of BsBacR (dark blue) and GtBacR (light green) is carried out and visualized by PyMOL 3.1.3. „N“ and „C“ indicate the N-terminus and C-terminus of the respective proteins.

**Figure S7: DNA binding of BsBacR.** Midoori Green-strained 10% native PAGE, using purified BacR from *B. subtilis* and a fragment of 1500 by containing the *yhdDEF* operon including the putative promoter.

**Figure S8: Squared displacement analysis (SQD) of BacR-mNG cells**. Upper panels in **A)** and **B)** show double or triple Rayleigh distributions to fit the observed data, N: number of trajectories generated by triplicates. The frequency of the diffusion constants (probability density) is shown as a function of the diffusion coefficient. The respective coefficient of determination (R²) is shown to assess the quality of the fit. In (B), the fit using three populations describes the underlying data better than the two-population fit in (A). Lower panels show corresponding quantile-quantile plots (Q-Q plots), illustrating the comparison between the measured (dashed line) and modeled (blue line) distributions for two (A) or three (B) populations.

**Figure S9: Cell fractionation analyses of BacR-mNG.** **A, C, D)** Western blot analysis of BacR-mNG (A treated with xylose; C treated with xylose or glucose), or of SecDF-mNG and PolA-mNG **(D)** after the cell fractionation process. The BacR-mNG (53.4 kDa), SecDF-mNG (108 kDa) and PolA-mNG (125.8 kDa) containing the mNeonGreen polypeptide (26.9 kDa). Fusion proteins were detected via mNG-Tag antibody. The fractions displayed are whole lysate, supernatant (Cytosol) and pellet (Membrane). **B, E)** Prior to western blotting, the total protein load of the gel was controlled by stain-free imaging, using Bio-Rad PROTEAN TGX Stain-Free SDS gels that allow for UV-activated quantitative imaging using UV exposure, before the gels are blotted onto a membrane. **F)** Western blot analysis of the quantity of BacR-mNG. Total cell extracts were used, the same amount of total protein was loaded per lane. **G)** Amido black staining used as a loading control of the prior western blot. The plot shows an example of the triplicate. **H)** Table of quantification of BacR-mNG levels. Standard deviation (SD) is calculated by triplicates.

**Figure S10: Localization of BacR-mNG under general stress, A)** salt stress (1.5 or 5 M NaCl) and **B)** ethanol stress (5 or 10%). Panels show bright field images (BF) or fluorescence images (BacR-mNG) of cells 30 minutes after addition of the stress.

**Figure S11: Localization pattern of BacR-mNG after antibiotic treatment.** **A)** Epifluorescence microscopy of BacR-mNG (cells growing with xylose). Cells were incubated for 30 min without (w/o) treatment, with vancomycin (van), ampicillin (amp), or erythromycin (ery). The chromosome was stained with DAPI. Merge shows the overlay of BacR-mNG and DAPI. Examples of the different localization patterns in every treatment are shown. The colors correspond to the legend at the top. Scale bar 2 μm.

**Table S1 oligonucleotides used in this study**

**Table S2 Plasmids used in this study**

References

1.	Letunic, I., and Bork, P. (2024) Interactive Tree of Life (iTOL) v6: recent updates to the phylogenetic tree display and annotation tool. *Nucleic Acids Res.* **52**, W78-W82
